# Elastomer-Mediated Interparticle Coupling Promotes Low-Threshold Rheological Network Formation in EPDM-g-MA-Compatibilized PP/Talc Composites

**DOI:** 10.3390/polym18192396

**Published:** 2026-09-30

**Authors:** Zeynep Keskin, Furkan Sarisoy, Gökçe Yilmaz, Zuhal Yurtbasi, Emine Kasgoz, Alper Kasgoz

**Affiliations:** 1 Polymer Materials Engineering Department, Faculty of Engineering, Yalova University, Yalova 77100, Turkey; keskinzeynep274@gmail.com (Z.K.); furkansarisooyy@gmail.com (F.S.); gkurt945@gmail.com (G.Y.); eminekasgoz@gmail.com (E.K.); 2 Polyshield Engineering Consultancy and R&D Co., Yalova 77100, Turkey

**Keywords:** polypropylene/talc composites, EPDM-g-MA, elastomer-mediated bridging, rheological percolation, compatibilization

## Abstract

Conventional compatibilization of polypropylene (PP)/mineral composites primarily improves local filler–matrix adhesion and dispersion. This study provides evidence for an elastomer-mediated interparticle bridging mechanism in which maleic anhydride-grafted ethylene propylene diene monomer (EPDM-g-MA) extends talc-associated polymer regions and promotes long-range elastic connectivity between neighboring platelets. Composites containing 5–40 wt% talc and 2.5–20 wt% EPDM-g-MA were characterized by frequency sweep, stress relaxation, strain sweep, and scanning electron microscopy. PP-g-MA and hybrid PP-g-MA/EPDM systems served as controls to distinguish conventional MA-mediated adhesion, independent elastomer addition, and their integration within EPDM-g-MA. PP-g-MA improved local interfacial continuity without promoting early network formation, whereas the hybrid systems failed to reproduce the low-threshold response of EPDM-g-MA. The apparent rheological network threshold decreased from approximately 40 wt% talc in the uncompatibilized system to approximately 30 wt% at 2.5–5 wt% EPDM-g-MA, 20 wt% at 10 wt%, and ≤5 wt% at 20 wt%. Effective interaction-volume analysis provides a geometric basis for the observed threshold reduction, while the combined rheological and morphological results support the development of talc-associated polymer regions into stress-bearing elastomer-mediated coupling. These findings establish compatibilizer architecture as a powerful strategy for controlling low-threshold melt-state network formation in PP/talc composites.

## 1. Introduction

Polypropylene (PP) is one of the most widely used semicrystalline thermoplastics in automotive, packaging, and consumer-goods applications because of its low density, balanced mechanical performance, ease of processing, and cost efficiency [1]. Nevertheless, the relatively low stiffness and limited dimensional stability of neat PP restrict its use in applications requiring greater rigidity and shape retention. Talc is therefore widely incorporated as a mineral filler because its platelet morphology improves stiffness, dimensional stability, and thermal resistance while maintaining acceptable processability [2,3,4,5]. Its nucleating activity and cost advantage further support its extensive industrial use, particularly in automotive PP compounds, which commonly contain approximately 5–20 wt% talc, although loadings of up to 40 wt% are employed in selected applications requiring enhanced rigidity and dimensional stability [1,2,3,4]. Despite these advantages, talc exhibits limited affinity for the apolar PP matrix because of its polar, hydroxylated surface. Hydroxyl-containing sites on the talc surface promote its hydrophilic character and can facilitate particle agglomeration in PP, making homogeneous dispersion and effective interfacial contact more difficult [6]. Accordingly, surface modification of talc has been explored to improve its compatibility with PP, and recent work has shown that silane–siloxane-based modification can enhance talc–PP interactions and improve the mechanical response of the resulting composites [7]. This polarity mismatch promotes interfacial debonding, platelet sliding, agglomeration, and localized stress concentration, thereby limiting stress transfer and the reinforcing efficiency of the filler [4,5,8,9]. Although talc does not necessarily induce macroscopic phase instability at moderate contents, the weak PP/talc interface restricts the formation of stable filler-associated structures [5,8,9]. In the melt state, this limitation is reflected in isolated or mechanically unstable particle contacts, weak long-range elastic continuity, and a high filler-content requirement for rheological network formation. Recent studies have also shown that elastomer-mediated surface reconstruction can substantially modify particle–matrix interfacial bonding and stress transfer in filled elastomeric systems [10].

Conventional strategies for overcoming this interfacial limitation include filler surface treatment, chemical modification of the polymer or filler, and the addition of compatibilizers that improve interfacial adhesion and dispersion. In PP/mineral composites, the most widely adopted approach is the use of PP-based compatibilizers containing polar functional groups. Peroxide-mediated radical functionalization provides another route for introducing reactive sites onto PP and enabling subsequent grafting or interfacial modification [11]. Maleic anhydride-grafted polypropylene (PP-g-MA) is the prevailing example because its PP backbone remains compatible with the matrix, while its anhydride groups interact with hydroxylated or ionic sites on mineral surfaces [4,5,11,12]. This architecture improves local filler–matrix adhesion, dispersion, and stress transfer and can therefore enhance stiffness and tensile performance [4,5,8,9,11]. However, PP-g-MA remains a conventional interfacial modifier: it strengthens the immediate particle–matrix boundary but does not inherently establish long-range elastic connectivity between spatially separated filler particles. The frequent addition of elastomeric modifiers, such as polyolefin elastomers or ethylene–propylene–diene rubber (EPDM), can improve deformation tolerance and partially restore the stiffness–toughness balance [13]. In such hybrid formulations, however, PP-g-MA and the elastomer perform separate functions: PP-g-MA governs local interfacial adhesion, whereas the elastomer primarily modifies matrix deformability. Their coexistence may alter the overall viscoelastic response, but it does not reproduce a covalently integrated elastomeric interphase in which polar functionality and a deformable backbone act within the same macromolecular architecture. Maleic-anhydride-grafted EPM has also been reported to improve phase compatibility and carbon-black distribution in NR/BR/EPDM blends [14]. More broadly, the effect of an elastomeric modifier depends strongly on its morphology and interfacial affinity. In situ nanofibrillation of EPDM altered the interfacial and rheological response of PP/EPDM composites while substantially improving their energy absorption and toughness [15]. Related rheological studies of polyolefin/elastomer blends have also demonstrated that elastomer content and morphology can substantially modify melt elasticity, relaxation behavior, and the resulting microstructure–property relationships [16]. More recently, Zhang et al. showed in PP/POE/talc composites that increasing the elasticity of the elastomer phase markedly enhanced the low-frequency elastic response, highlighting the importance of elastomer viscoelasticity in governing melt-state structural behavior [17].

Of particular relevance to mineral-filled PP, SEBS-g-MA encapsulated and disoriented talc platelets more extensively than unmodified SEBS, illustrating how graft architecture can redirect elastomer localization at the filler interface [18].

Rheological percolation provides a sensitive means of distinguishing local interfacial improvement from the development of long-range connectivity in filled polymer melts. Below the percolation threshold, the viscoelastic response remains predominantly matrix-controlled, while filler particles impose only isolated or transient constraints that cannot sustain stress over long time scales. Once a continuous, system-spanning structure develops, polymer relaxation becomes increasingly restricted and the terminal response shifts toward elastic dominance. This transition is commonly identified by the emergence of a low-frequency storage-modulus plateau, suppression of the terminal frequency dependence of G′, and a frequency-independent or decreasing low-frequency tan δ response [19,20,21,22,23,24,25,26,27]. In conventional filled-polymer systems, such behavior generally requires sufficiently high filler contents for direct contacts or closely spaced interparticle interactions to become persistent, particularly for high-aspect-ratio fillers such as talc [28]. The resulting network can enhance low-frequency elasticity, long-time stress relaxation, and resistance to flow-induced structural relaxation. However, when connectivity is governed predominantly by a densely packed rigid-filler skeleton, the same network may become increasingly strain-sensitive and undergo early nonlinear breakdown [29,30,31], with possible consequences for the stiffness–deformation balance of the final material. The processing significance of rheological percolation therefore depends not only on whether a network forms, but also on the physical nature of the connections that sustain it. A network supported by deformable polymer-mediated links may maintain long-range elastic continuity while permitting greater local stress redistribution than one governed primarily by rigid particle–particle contacts [29,30,32,33,34,35,36]. Rheology is particularly suitable for examining this distinction because it probes both the emergence of long-range melt-state connectivity and the stability of the resulting structure under prolonged and increasing deformation.

Previous studies therefore establish that elastomeric modifiers and graft architecture can influence filler localization, interfacial organization, and the rheological response of polymer/filler systems [14,15,16,18]. However, these studies do not resolve whether compatibilizer molecular architecture can systematically control the rheological percolation threshold of PP/talc composites. In particular, this question has not been directly examined by separating MA-mediated interfacial modification, independent elastomer addition, and the covalent integration of both functions within a single grafted elastomer. Rather than introducing a separate elastomeric phase, the present study targets the architecture of the PP/talc interfacial region using EPDM-g-MA. Its MA functionalities may promote interfacial association with hydroxylated sites on the talc surface, while the flexible EPDM backbone extends into the polymer-rich phase [5,11,13]. Similar effects have been reported in maleic-anhydride-functionalized PP/talc systems, where changes in melt rheology and morphology were attributed to stronger filler–polymer interactions [37,38,39]. In ternary PP/elastomer/talc systems, elastomer polarity has also been shown to influence preferential association with and coating of talc surfaces [40]. Spectroscopic studies on related maleic-anhydride-functionalized polyolefin/hydroxylated-mineral systems further indicate that MA functionalities can participate in specific polar interactions with surface hydroxyl groups [41]. However, the present data do not establish the specific molecular nature of the EPDM-g-MA/talc interaction. Increasing EPDM-g-MA content may therefore enlarge the effective talc-associated polymer region and increase the probability of overlap between neighboring regions.

Such overlap could provide deformable, stress-bearing interparticle coupling without requiring direct talc–talc contact. Chain interpenetration and entanglement provide a plausible molecular basis for this behavior [32,33,34,35,36], although these processes are not directly resolved in the present study.

This study tests this hypothesis using oscillatory frequency sweep, stress relaxation, strain sweep, scanning electron microscopy, and effective interaction-volume analysis. EPDM-g-MA and talc contents were systematically varied, with the compatibilizer range extended to 20 wt% as a mechanistic probe rather than as a proposed practical optimum. PP-g-MA formulations were used to determine whether MA-mediated interfacial modification alone could reproduce the response, whereas PP-g-MA/EPDM systems were used to test whether the separate coexistence of MA functionality and an elastomeric phase was sufficient. This comparative design therefore isolates the contribution associated with their covalent integration within EPDM-g-MA. Accordingly, the novelty of the present study lies in demonstrating architecture-dependent control of the apparent rheological percolation threshold, rather than in the general concept of elastomer-mediated particle bridging. The observed distinction is therefore interpreted in terms of compatibilizer architecture rather than a presumed higher intrinsic thermodynamic affinity of EPDM-g-MA toward talc.

## 2. Materials and Methods

### 2.1. Materials

The matrix polymer used in this study was a polypropylene homopolymer, Moplen HP401R, supplied by LyondellBasell (Dubai, United Arab Emirates). The material has a melt flow rate of 25 g/10 min (230 °C/2.16 kg) and was used as received. Talc was supplied by Kimyalab (Istanbul, Turkey) and used without further treatment. According to the supplier data, the talc had a median particle size of 4.52 µm, a D97 value of 23.7 µm, a fraction below 2 µm of 38%, and a density of 2.73 g cm^−3^. Maleic anhydride-grafted EPDM (EPDM-g-MA, grade PL5110) supplied by Polyalloy (Taoyuan City, Taiwan) was employed as the primary compatibilizer. According to supplier specifications, the nominal maleic anhydride grafting level of EPDM-g-MA is approximately 1 wt%. For reference purposes, maleic anhydride-grafted polypropylene (PP-g-MA, grade PL1010, Polyalloy) and unmodified EPDM (Vistalon 722) supplied by ExxonMobil (Spring, TX, USA) were also used in selected formulations. The PP-g-MA grade also contains approximately 1 wt% maleic anhydride, allowing comparison at comparable nominal MA contents. The supplier-reported melt flow indices of EPDM-g-MA (PL5110) and PP-g-MA (PL1010) are 0.4 and 80 g/10 min, respectively (190 °C/2.16 kg). The unmodified EPDM (Vistalon 722) has a melt index of 1.0 g/10 min under the same conditions.

### 2.2. Sample Preparation

All formulations were melt-compounded using a co-rotating twin-screw extruder (Kökbir RTX-T18, Kırklareli, Turkey; L/D = 25). PP, talc, and the compatibilizers were dry-mixed prior to extrusion to ensure uniform feeding. The barrel temperature profile was set to 30–60–170–180–190–210 °C from the feed zone to the die, and the screw speed was maintained at 200 rpm. The extrudates were cooled under ambient air conditions and subsequently pelletized by granulation. The resulting pellets were used directly for rheological characterization and represent the final compounded materials. A systematic sample notation was adopted to enable consistent comparison among uncompatibilized, grafted, and hybrid formulations. Unmodified PP/talc composites were designated as T5, T10, T20, T30, and T40, corresponding to talc loadings of 5–40 wt%. Formulations containing EPDM-g-MA were denoted as xMA–Ty, where x represents the EPDM-g-MA content (2.5, 5, 10, or 20 wt%) and Ty indicates the talc loading (wt%). For PP-g-MA-based formulations, the same notation was used, denoted as xPPMA–Ty to reflect the PP-g-MA content (x) and the corresponding talc loading (Ty). Hybrid formulations containing both PP-g-MA and unmodified EPDM were denoted using a combined notation. In these systems, samples labeled as 2.5PPMA–2.5EPDM–Ty and 20PPMA–20EPDM–Ty indicate formulations in which PP-g-MA and EPDM were added at equal weight fractions (2.5 or 20 wt%, respectively), while Ty represents the talc loading.

In all formulations, the stated talc, compatibilizer, and/or EPDM contents are expressed as wt% of the total formulation, while PP constitutes the balance to 100 wt%. For example, 10MA–T20 contains 10 wt% EPDM-g-MA, 20 wt% talc, and 70 wt% PP, whereas 20PPMA–20EPDM–T20 contains 20 wt% PP-g-MA, 20 wt% EPDM, 20 wt% talc, and 40 wt% PP.

### 2.3. Characterizations

Rheological characterizations were performed using a rotational rheometer (Discovery Hybrid Rheometer-3, DHR-3, TA Instruments, New Castle, DE, USA) equipped with a 25 mm parallel-plate geometry. The measurement gap was set to 1000 µm. Three complementary rheological tests—frequency sweep, stress relaxation, and strain sweep—were employed to characterize the melt-state viscoelastic behavior and structural evolution of the composites. Prior to frequency-sweep measurements, the linear viscoelastic region (LVR) of each formulation was determined by preliminary strain–sweep tests conducted over a strain range of 0.01–10% at a fixed angular frequency of 10 rad s^−1^. These scans were used solely to identify the strain interval in which the response remained linear. All subsequent frequency-sweep tests were performed at a constant strain selected within the LVR for each sample. Frequency-sweep measurements were carried out over an angular frequency range of 600 to 0.1 rad s^−1^. Stress-relaxation tests were performed under a constant strain of 0.1%, and the time-dependent relaxation modulus E(t) was monitored for 300 s to investigate long-time relaxation behavior and the stability of melt-state microstructures.

Oscillatory strain–sweep measurements were subsequently performed on the rotational rheometer at 200 °C over a strain range of 0.1–100% and at a fixed angular frequency of 10 rad s^−1^. The evolution of G′ with increasing strain was analyzed to determine the plateau modulus, G_p_′, and the critical strain associated with the onset of nonlinear viscoelasticity. G_p_′ was calculated as the mean G′ value over the strain-independent region preceding the onset of modulus decay. The critical strain, γc, was defined as the strain at which G′ decreased by 10% relative to G_p_′, marking the onset of nonlinear viscoelasticity and deformation-induced structural breakdown [29,30,31,42]. All rheological tests were conducted at 200 °C. For each measurement, a fresh specimen was used, and samples were allowed to equilibrate at the test temperature for 5 min prior to data acquisition to ensure thermal and stress relaxation.

The fracture-surface morphology of the selected composites was examined using a scanning electron microscope (ZEISS Sigma 300 FE-SEM, Carl Zeiss AG, Oberkochen, Germany). The specimens were fractured under liquid nitrogen to expose the internal morphology and were subsequently coated with a thin layer of gold. Images were collected at multiple magnifications to evaluate talc dispersion, interfacial gaps, platelet pull-out, and the continuity of the polymer morphology surrounding the talc platelets.

In addition to the morphological characterization, component volume fractions and upper-bound effective interaction volumes were calculated to support the mechanistic interpretation of network formation. The volume fraction of each component was calculated from its weight fraction and density according to

(1) $$\phi_{i}=\frac{w_{i}/\rho_{i}}{\sum_{j}w_{j}/\rho_{j}}$$

where $w_{i}$ and $\rho_{i}$ represent the weight fraction and density of component i, respectively. Densities of 0.905, 2.73, and 0.87 g cm^−3^ were used for PP, talc, and EPDM-g-MA, respectively. For the percolation analysis, the corresponding talc volume fractions of the critical compositions were calculated from the nominal formulations using Equation (1). The talc volume fraction of the uncompatibilized T40 composition was taken as the reference critical volume fraction associated with the apparent rheological network transition. To estimate the maximum geometric contribution of the talc-associated EPDM-g-MA region, an upper-bound effective interaction volume was calculated by assigning the entire EPDM-g-MA volume to the talc-associated region:

(2) $$\phi_{int,max}=\frac{w_{talc}/\rho_{talc}+w_{EPDM-g-MA}/\rho_{EPDM-g-MA}}{\sum_{j}w_{j}/\rho_{j}}$$

## 3. Results and Discussion

### 3.1. Interfacial Structuring and Rheological Transitions in EPDM-g-MA Compatibilized PP/Talc Composites

Before interpreting the frequency-dependent rheological response, rheological stability over the experimental time scale was verified by time-sweep measurements at 200 °C for 1800 s using representative materials selected to cover the polymer matrix, elastomeric and grafted components, and characteristic talc-containing formulations of the system. As shown in Figure S2, G′ remained essentially stable throughout the measurement period for all investigated samples, with no systematic time-dependent decrease in modulus. These results indicate that progressive thermal degradation did not significantly influence the rheological response over the experimental time scale used in this study.

**Figure 1:**
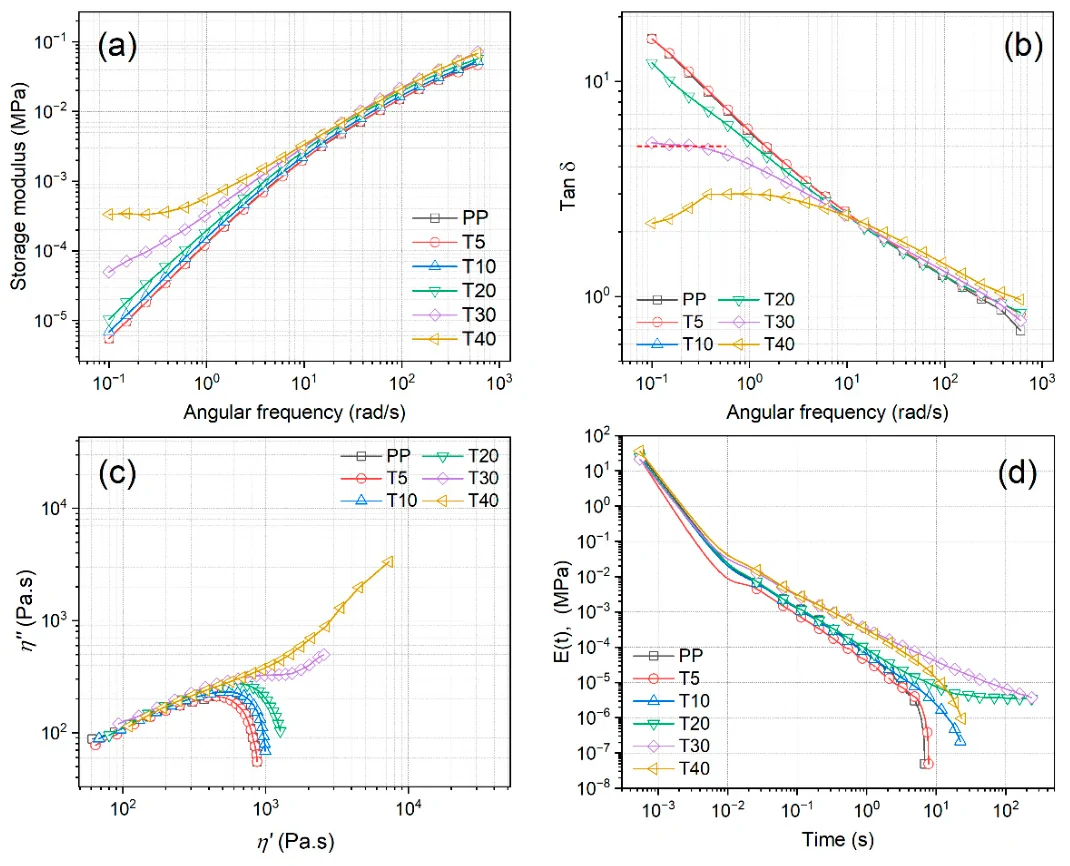
Rheological characterization of uncompatibilized T-series composites: (**a**) G′ versus ω; (**b**) tan δ versus ω; (**c**) Cole–Cole plot of η″ versus η′; (**d**) E(t) versus time.

The rheological evolution of the PP/talc composites was first examined using the uncompatibilized T-series as a reference. This baseline was then used to evaluate how increasing EPDM-g-MA content modifies the transition from isolated filler-induced constraints to network-like elastic connectivity. Figure 1 presents an overview of the viscoelastic response of uncompatibilized PP/talc composites as a function of talc content. The results include (a) G′ versus angular frequency (ω), (b) tan δ versus ω, (c) Cole–Cole plots of η″ versus η′, and (d) time-dependent relaxation modulus from stress relaxation tests in the melt state. As shown in Figure 1a, G′ decreases monotonically with decreasing angular frequency for all compositions, although the extent of this frequency dependence becomes progressively weaker at higher talc loadings. Up to 20 wt% talc (T20), the G′ curves largely follow the same decay trend as neat PP, consistent with weak or disconnected filler-induced microstructures that are insufficient to sustain long-term stress in the low-frequency regime. At 30 wt% talc (T30), G′ begins to deviate from this behavior, exhibiting a clear upward shift at low angular frequencies together with a reduction in the terminal slope from 1.271 for T20 to 0.802 for T30. However, this deviation remains limited compared with the abrupt separation observed at 40 wt% talc, indicating that T30 represents an intermediate state rather than a fully developed elastic network. In T40, G′ displays a well-defined plateau in the terminal region, with the terminal slope decreasing further to 0.205, indicating the emergence of an elastic contribution that no longer depends strongly on frequency. This behavior marks a transition toward a more structured, elastically dominated response, consistent with the development of a percolated filler network—a continuous, system-spanning structure that transforms the melt from a liquid-like to a solid-like viscoelastic state once a critical filler content is exceeded. However, since G′ alone cannot unambiguously confirm the existence of a fully percolated structure, additional insight can be obtained by analyzing the variation in tan δ (G″/G′) with angular frequency. This approach offers improved sensitivity to the balance between elastic and viscous contributions, particularly in the low-frequency regime, and enables a more refined assessment of filler network formation. In this context, Figure 1b illustrates the evolution of tan δ as a function of angular frequency for uncompatibilized PP/talc composites. In line with the G′ trends, all samples up to T20 exhibit frequency-dependent damping characteristics similar to neat PP, indicating that filler-induced network effects are not yet significant. However, the T30 sample deviates markedly, with the low-frequency tan δ slope changing from −0.364 for T20 to −0.093 for T30. According to the Winter–Chambon criterion, the gel point of a viscoelastic system is characterized by identical power-law scaling of G′ and G″, which leads to a frequency-independent tan δ [19,20]. The near-zero slope of T30 therefore indicates that this composition approaches the critical regime but has not yet developed a fully percolated structure. In T40, the slope changes further to +0.158, indicating a clear departure from the matrix-dominated terminal response and supporting its classification as a post-critical rheologically connected composition. Therefore, the low-frequency slope of tan δ not only supports the identification of the percolation threshold in the uncompatibilized PP/talc series but also provides a baseline criterion for evaluating how different compatibilizer architectures shift network formation in the following sections.

The Cole–Cole plots in Figure 1c provide further insight into the relaxation-time distribution of the uncompatibilized PP/talc composites. Up to 20 wt% talc (T20), the curves display nearly perfect semi-circular shapes, consistent with a relatively homogeneous relaxation spectrum dominated by the PP matrix [24,27,42]. This observation suggests that talc does not significantly disturb the relaxation-time distribution of the melt within this concentration range, in agreement with the G′ and tan δ results. At higher talc loadings, the Cole–Cole response begins to deviate from this matrix-dominated behavior. The T30 sample shows the emergence of a low-frequency tail extending from the primary arc, whereas T40 exhibits a more pronounced, extended tail. Such tail formation is commonly associated with filler-induced microstructural constraints, where talc particles suppress the long-time relaxation modes of the polymer matrix, and should not be misinterpreted as evidence of a separate incompatible phase [24,27,42]. Rather, the tail is consistent with an additional elastic-like relaxation contribution arising from restricted segmental mobility near the talc surface. This interpretation is consistent with the G′ and tan δ analyses, which identified T30 as a near-critical composition and T40 as having reached rheological percolation.

The stress relaxation results for the uncompatibilized PP/talc composites are shown in Figure 1d. Neat PP exhibits the expected liquid-like response, with E(t) progressively approaching zero at long times. At low filler contents, no stable reinforcement is achieved; T5 relaxes even faster than the matrix, which is consistent with weak filler–matrix coupling and possible interfacial slip, while T10 provides only minor stiffening but ultimately follows the same liquid-like relaxation trend. The T20 composite represents the last concentration that still retains a largely polymer-like relaxation profile, showing intermediate-time behavior nearly identical to neat PP. Nevertheless, the presence of talc slows the long-time decay and produces a pronounced stress-retention region, although a fully permanent elastic plateau is not established. This apparent difference from the frequency-sweep, tan δ, and Cole–Cole results is not contradictory, since stress relaxation probes longer time scales and can therefore reveal weak or incipient talc–talc contacts that remain less evident under small-amplitude oscillatory conditions [27,42]. A distinct change occurs at T30, where the relaxation curve adopts a more gradual, nearly linear decay at long times. Although the stress relaxation data alone do not prove network formation, the complementary frequency-sweep and tan δ analyses indicate that this composition lies close to the critical regime, where localized network domains may begin to emerge. Finally, T40 exhibits the strongest deviation, retaining the highest modulus at short-to-intermediate times but undergoing an abrupt stress collapse at long times. Although the G′ and tan δ results support the classification of T40 as a rheologically percolated composition under small-amplitude oscillatory conditions, its inability to maintain a stable stress response during prolonged relaxation indicates that percolation and network stability should be considered separately. In this sense, T40 exhibits rheological signatures of system-spanning connectivity, whereas the localized network domains in T30 appear to provide a more stable stress-bearing response over the relaxation window, despite not forming a fully percolated structure. This behavior is consistent with the limited stability of the uncompatibilized talc network in the PP matrix. The apolar PP matrix interacts poorly with the hydroxylated talc surface [4,5,8,9], which may favor weak van der Waals and frictional contacts rather than strong interfacial anchoring. In addition, talc platelets can undergo basal-plane slip [43], which may allow filler–filler contacts to unjam under sustained deformation. As a result, even though T40 exhibits a continuous-network-like rheological response, the network is unable to fully carry stress in the melt over long times and instead may relax through interfacial sliding and structural yielding [29,30,31].

**Figure 2:**
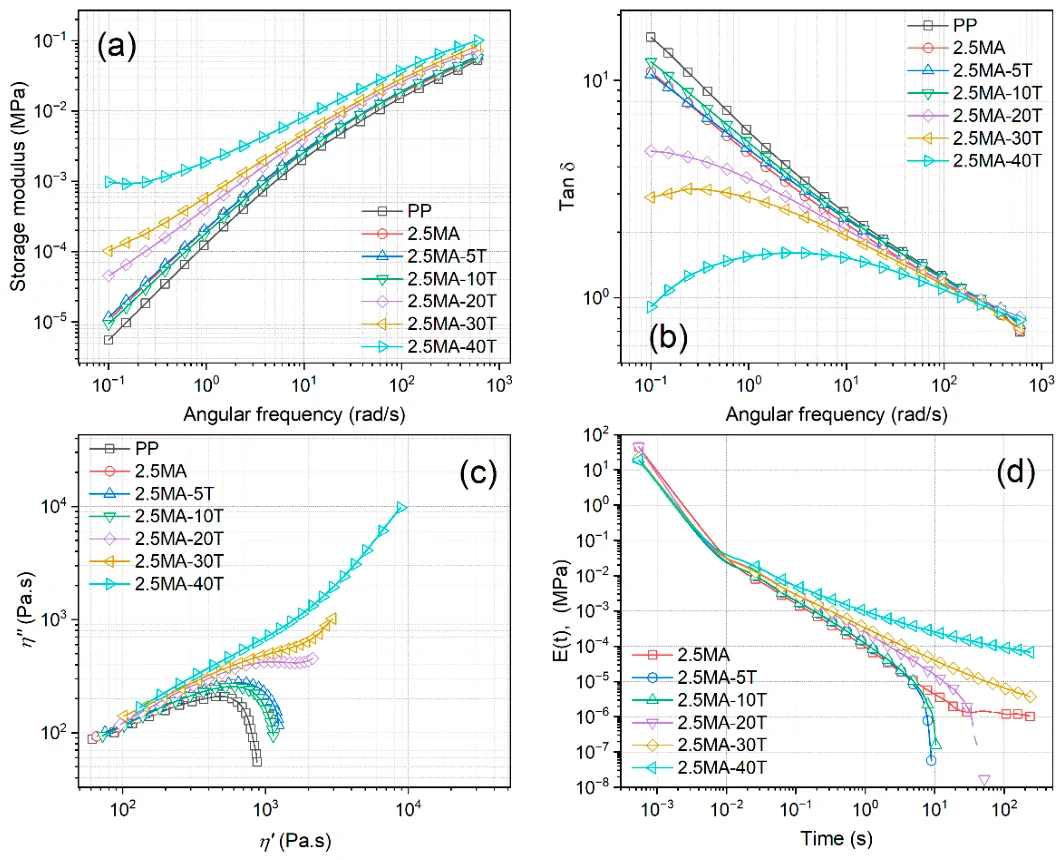
Rheological characterization of 2.5MA-series composites: (**a**) G′ versus ω; (**b**) tan δ versus ω; (**c**) Cole–Cole plot of η″ versus η′; (**d**) E(t) versus time.

**Figure 3:**
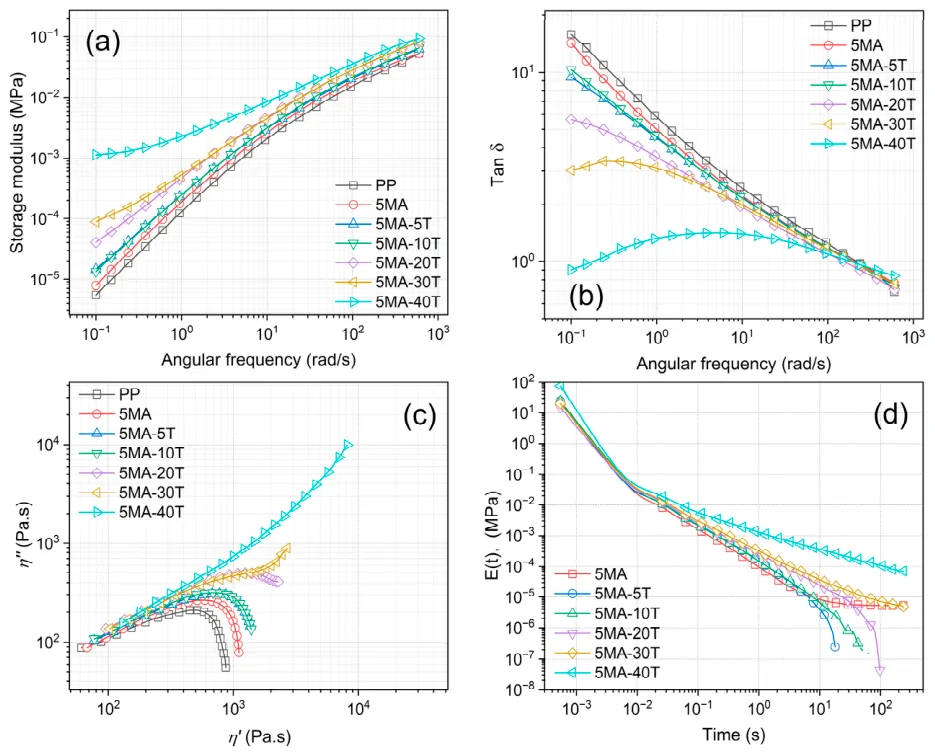
Rheological characterization of 5MA-series composites: (**a**) G′ versus ω; (**b**) tan δ versus ω; (**c**) Cole–Cole plot of η″ versus η′; (**d**) E(t) versus time.

In the high angular frequency region (≈600 rad s^−1^), both the 2.5MA and 5MA series exhibit higher G′ values than their uncompatibilized counterparts at the same talc loading (Figure 1a, Figure 2a and Figure 3a). For example, at 40 wt% talc, G′ increases from approximately 68.6 kPa for T40 to 100.6 and 98.1 kPa for 2.5MA–T40 and 5MA–T40, respectively, which is consistent with enhanced filler–matrix interfacial interactions under rapid deformation [4,5,11]. At low angular frequencies, both series also exhibit elevated G′ values compared to neat PP and their uncompatibilized counterparts, which is consistent with the additional elastic contribution of the EPDM-g-MA phase together with improved interfacial adhesion [11,13]. For example, at ω = 0.1 rad s^−1^, G′ increases from approximately 0.3 kPa for T40 to about 1.0 kPa for 2.5MA–T40, corresponding to an increase of more than threefold. Increasing the EPDM-g-MA content to 5 wt% produces little further change, with 5MA–T40 remaining within the same approximate modulus range. This initial increase, followed by the comparable response of the 2.5MA and 5MA systems, indicates that the effect becomes nearly saturated within this low-compatibilizer range. In the absence of a compatibilizer, the composite containing 30 wt% talc (T30) exhibits signs of localized network formation, whereas rheological signatures consistent with full percolation are observed only at 40 wt% talc (T40). With the incorporation of 2.5 wt% EPDM-g-MA, the 2.5MA-T30 sample displays a marked increase in low-frequency G′, while the terminal slope decreases from 0.964 for 2.5MA-T20 to 0.765 for 2.5MA-T30 (Figure 2a), suggesting a shift in the network transition toward lower talc content. A similar response is observed in the 5MA series, where increasing the compatibilizer content from 2.5 to 5 wt% does not produce a further clear reduction in the apparent threshold (Figure 3a). Since G′ alone does not unambiguously confirm a fully percolated structure, the apparent position of the percolation threshold is further assessed using the tan δ response discussed below.

Following the same criterion used for the uncompatibilized T-series, the low-frequency slope of tan δ was used to distinguish between localized connectivity and fully developed network formation in the 2.5MA and 5MA series [19,20,27]. For 2.5MA-T20, the tan δ curve exhibits a negative low-frequency slope (−0.125), indicating that the terminal response remains pre-critical. At 2.5MA-T30, the slope approaches zero (+0.001), indicating an approximately frequency-independent tan δ response consistent with the critical rheological regime. At 2.5MA-T40, the slope becomes positive (+0.231), indicating progression beyond the critical condition toward a more elastic-dominated terminal response (Figure 2b). Increasing the EPDM-g-MA content from 2.5 to 5 wt% does not further reduce the apparent percolation threshold. In the 5MA series, 5MA-T20 still reflects localized interactions, whereas 5MA-T30 and 5MA-T40 exhibit low-frequency tan δ responses consistent with percolated structures (Figure 3b). Therefore, the tan δ analysis indicates that both 2.5MA and 5MA shift the apparent percolation threshold from 40 wt% talc in the uncompatibilized PP/talc system to approximately 30 wt% talc, while increasing the compatibilizer content from 2.5 to 5 wt% produces only limited further change in network formation.

As shown in the Cole–Cole plots (Figure 2c and Figure 3c), the addition of talc to EPDM-g-MA–based blends leads to both a broadening of the arcs and an extension of the low-frequency tail regions, consistent with a more heterogeneous viscoelastic microstructure [24,27,42]. With increasing filler content, the arc diameter along the η′ axis and the η″ magnitude increase, suggesting a wider distribution of relaxation times and intensified dissipative contributions. Tail formation becomes evident already in the compatibilized systems containing 20 wt% talc, particularly in 2.5MA-T20 and 5MA-T20, consistent with the emergence of long-τ relaxation modes associated with localized network domains [24,27,42]. At higher talc contents, especially 30–40 wt%, the tails become more pronounced and extend further than in the corresponding uncompatibilized composites, indicating more pronounced long-time relaxation constraints in the compatibilized systems. This elongation of the low-frequency tails supports the tan δ-based conclusion that the apparent percolation threshold shifts from 40 wt% in the uncompatibilized system to approximately 30 wt% in the EPDM-g-MA compatibilized blends.

In the PP/talc composites compatibilized with 2.5 and 5 wt% EPDM-g-MA, the stress relaxation behavior is illustrated in Figure 2d and Figure 3d. In these systems, the relaxation modulus E(t) initially decays rapidly but then diverges into composition-dependent trends at longer times. Talc-free PP/EPDM-g-MA blends (2.5MA and 5MA) exhibit a partial stress-retention region at later stages, consistent with the sustained elastic contribution of the rubbery phase even in the absence of a filler network [13,27,42]. By contrast, the talc-filled composites with 5 and 10 wt% talc (2.5MA-T5, 2.5MA-T10, 5MA-T5, and 5MA-T10) show rapid relaxation and early loss of stress retention. This behavior is consistent with weak, discontinuous talc domains that act as local stress concentrators rather than effective reinforcing structures [8,9,29]. In these compositions, the talc content appears insufficient to support a connected network, and the presence of EPDM-g-MA does not yet provide enough interparticle connectivity to stabilize the long-time relaxation response. At 20 wt% talc (2.5MA-T20 and 5MA-T20), the relaxation curves become more gradual at long times, consistent with localized structural connectivity that temporarily delays stress relaxation. However, these structures do not yet produce a fully stable, system-spanning network. At 30 and 40 wt% talc (2.5MA-T30, 2.5MA-T40, 5MA-T30, and 5MA-T40), the stress relaxation response becomes more persistent and follows a nearly linear, power-law-like decay without abrupt collapse, supporting the presence of a rheologically connected structure [19,20,27]. Importantly, while the talc-only T40 blend eventually lost its load-bearing capacity and exhibited stress collapse, the EPDM-g-MA compatibilized T40 systems (2.5MA-T40 and 5MA-T40) maintained stress over the entire relaxation window, indicating a substantial improvement in network durability and elastic retention. Compared to their talc-only counterparts, the compatibilized systems display not only more persistent relaxation but also rheological signatures consistent with greater structural stability over long timescales.

**Figure 4:**
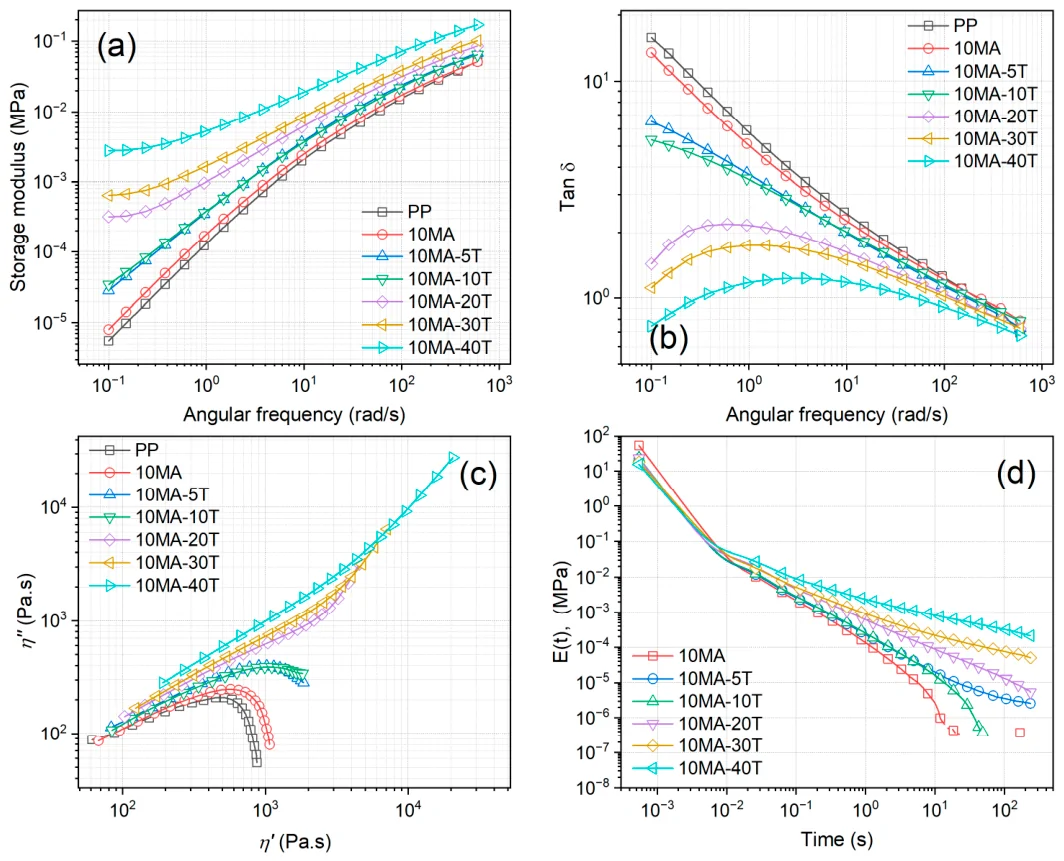
Rheological characterization of 10MA-series composites: (**a**) G′ versus ω; (**b**) tan δ versus ω; (**c**) Cole–Cole plot of η″ versus η′; (**d**) E(t) versus time.

**Figure 5:**
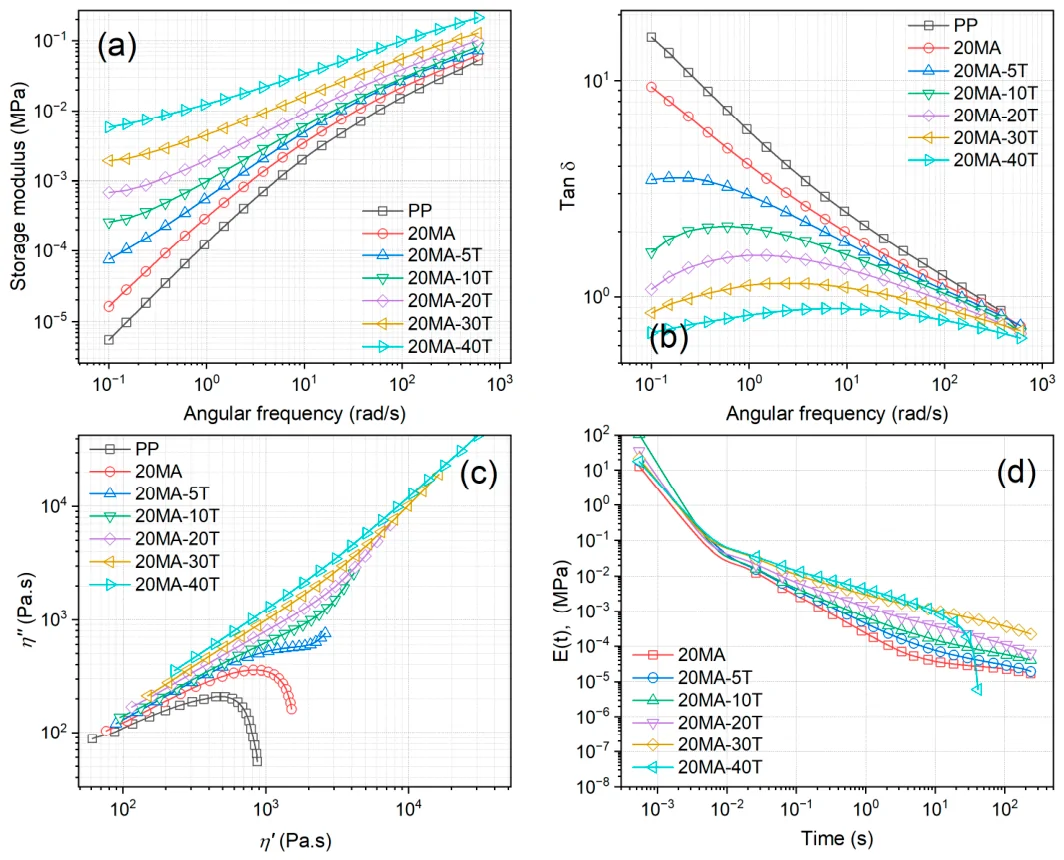
Rheological characterization of 20MA-series composites: (**a**) G′ versus ω; (**b**) tan δ versus ω; (**c**) Cole–Cole plot of η″ versus η′; (**d**) E(t) versus time.

In the high angular frequency region (≈600 rad s^−1^), the talc-free PP/EPDM-g-MA references exhibit high-frequency G′ values that remain very close to neat PP, indicating that increasing the compatibilizer content alone does not produce a significant modulus enhancement at this deformation rate (Figure 4a and Figure 5a). As shown in Figure 4a and Figure 5a, introducing talc produces a sharp increase in high-frequency G′ for the 10MA and 20MA composites, in clear contrast to both the talc-free references and the nearly unchanged response observed between the 2.5MA and 5MA series. This demonstrates that pronounced high-frequency stiffening emerges only when the compatibilizer content reaches 10 wt% and is combined with talc. The magnitude of this rise becomes especially evident at higher filler levels, as reflected by the pronounced enhancement observed in the 10MA-T40 sample. In the PP/talc composites containing 20 wt% EPDM-g-MA (Figure 5a), the high-frequency modulus increases steadily with talc content, although the difference between the 10MA and 20MA formulations at identical filler loadings remains moderate rather than dramatic. This indicates that the primary stiffening effect of the compatibilizer is already largely established at 10 wt%, and the additional increase to 20 wt% mainly reinforces the existing trend rather than producing a new jump in modulus.

In the low-frequency regime, the differences between formulations become more pronounced and provide clearer insight into long-range structural behavior. Unlike the 2.5MA and 5MA talc-free references, which remain close to neat PP across the whole frequency range, the talc-free 10MA blend shows only a slight low-frequency increase, whereas the 20MA blend displays a distinctly higher modulus, indicating that EPDM-g-MA begins to contribute an intrinsic elastic component to the melt at higher compatibilizer contents even without filler. Once talc is introduced, both series exhibit a more pronounced increase in low-frequency G′ relative to their corresponding talc-free references. The PP/talc composites containing 10 wt% EPDM-g-MA show a clear increase in G′ relative to neat PP, and this increase becomes more pronounced when the compatibilizer content is raised to 20 wt% EPDM-g-MA. In this frequency range, the combined evaluation of both the modulus level and the terminal-region slope provides a clear distinction between the 10MA and 20MA series. In the 10 wt% EPDM-g-MA composites, the low-frequency G′ increases with talc loading, while the terminal slope remains relatively high at 5 and 10 wt% talc, with values of 1.115 and 1.048, respectively. A decisive change appears at 20 wt% talc, where the terminal slope decreases to 0.507 and G′ at 0.1 rad s^−1^ rises by approximately one order of magnitude, indicating the onset of network formation in this series [19,20,21,22,23,24,25,26,27]. In contrast, the 20MA composites show both a higher low-frequency modulus and a much earlier reduction in the terminal slope. The G′ terminal slope is already reduced to 0.881 at 5 wt% talc and decreases further to 0.590 at 10 wt% and 0.460 at 20 wt%, confirming that the terminal response becomes progressively flatter at substantially lower talc contents in this series. These features align with the higher overall modulus levels observed in Figure 5a and demonstrate that the approach toward a frequency-independent terminal response occurs at substantially lower talc contents when the compatibilizer level is increased to 20 wt%. Since G′ alone does not unambiguously confirm a fully percolated structure, the apparent percolation thresholds for the 10MA and 20MA series are further assessed using the tan δ response discussed below.

The tan δ responses provide a complementary view of the viscoelastic behavior and reinforce the distinctions between the 10MA and 20MA series (Figure 4b and Figure 5b). In the 10MA composites, tan δ remains above unity across most talc loadings, indicating that viscous dissipation continues to dominate the terminal region. A noticeable change appears only between 10 and 20 wt% talc, where the low-frequency tan δ slope changes from −0.184 to +0.174, signaling a transition from local structuring toward the onset of network formation. This trend is consistent with the one-order-of-magnitude rise in low-frequency G′ and the nearly horizontal terminal response observed at 20 wt% talc in Figure 4a, together pointing to a percolation threshold at around 20 wt% talc for this series. In the 20MA series, the effect of talc becomes considerably more pronounced. The low-frequency tan δ slope changes from −0.365 for the talc-free 20MA reference to −0.068 at only 5 wt% talc, indicating that the terminal response approaches frequency independence at a much earlier stage than in the 10MA composites. More importantly, at higher talc loadings, particularly 30 and 40 wt%, tan δ approaches or falls below unity in the low-frequency region, showing that the elastic contribution becomes comparable to or greater than viscous dissipation. This behavior distinguishes the 20MA series from the 10MA composites, where tan δ remains above unity despite the onset of network formation. This early suppression of the slope, together with the lower absolute tan δ values than in the 10MA series and the higher low-frequency G′ values shown in Figure 5a, demonstrates that increasing the compatibilizer content strengthens the influence of talc to the point where network-like behavior emerges at significantly lower filler loadings. Taken together, the low-frequency G′ and tan δ responses show that the apparent rheological network threshold decreases systematically from approximately 40 wt% talc in the uncompatibilized system to 30 wt% at 2.5–5 wt% EPDM-g-MA, 20 wt% at 10 wt% EPDM-g-MA, and ≤5 wt% at 20 wt% EPDM-g-MA.

The apparent percolation thresholds identified in the previous section reveal a trend that runs counter to the usual expectation for filled polymer systems. A system that becomes better dispersed and more strongly bonded at the interface would normally be expected to reach network formation only at higher, or at least similar, filler contents, because improved particle separation increases the mean interparticle distance [25,32,33,36]. Contrary to this expectation, the apparent percolation threshold in the present system does not increase with compatibilizer content. Instead, it progressively shifts to lower talc contents, far below the threshold observed for the uncompatibilized PP/talc system and below the values typically expected for conventional PP/talc composites containing plate-like mineral fillers [2,3,4,5,21,22,24]. Most strikingly, the 20MA series exhibits network-like behavior at 5 wt% talc (1.69 vol%), the lowest loading examined, placing the apparent network threshold at ≤5 wt% talc. This marks the onset of elastomer-mediated connectivity at a filler content well below the level expected from the volume occupied by the bare talc platelets alone. Based on the particle size of the talc used in this work, direct talc–talc contact alone is unlikely to fully account for the observed network-like response at such low loadings [32,33,34,35,36], even under idealized assumptions. This geometric limitation is examined quantitatively through the effective interaction-volume analysis in Section 3.5. Such a pronounced threshold reduction therefore points to an interaction between EPDM-g-MA and talc that goes beyond conventional compatibilization. The evolution of the percolation threshold with compatibilizer content provides further insight into this mechanism.

The first reduction in threshold, from 40 wt% talc (18.0 vol%) in the uncompatibilized system to 30 wt% talc (≈12.3 vol%) at 2.5 wt% EPDM-g-MA, is consistent with the initial development of a talc-associated polymer region that extends the effective interaction range of the platelets. Increasing the compatibilizer content to 5 wt% does not further lower the threshold. Although the talc-associated region may continue to grow with increasing compatibilizer content, at these loadings it appears insufficient to establish mechanically effective overlap between neighboring talc-associated regions, and the threshold remains at 30 wt% talc. This plateau in the threshold is significant. Rather than indicating that interfacial-region growth has ceased, the unchanged threshold between 2.5 and 5 wt% EPDM-g-MA may reflect a composition range in which the effective interaction volume increases without yet reaching the overlap condition required for persistent interparticle connectivity. At higher EPDM-g-MA contents, continued growth of the talc-associated regions progressively extends the interaction range of the platelets and increases the probability of overlap between neighboring regions. Beyond approximately 10 wt% EPDM-g-MA, the threshold decreases again from 30 wt% talc (≈12.3 vol%) to 20 wt% talc (7.57 vol%), and then drops more dramatically to ≤5 wt% talc (≤1.69 vol%) at 20 wt% EPDM-g-MA. This renewed and intensified reduction is interpreted in terms of the transition from interfacial-region growth to mechanically functional elastomer-mediated coupling.

In this proposed mechanism, the maleic anhydride functionalities may promote interfacial association of EPDM-g-MA with polar sites on the talc surface [37,38,39,40,41], potentially allowing talc-associated chains or polymer regions associated with neighboring platelets to interact across the interparticle space. The elastomer backbone may therefore act as a flexible connector that couples adjacent platelets even when they are not in direct contact. Such chain-mediated coupling may restrict the mobility of surrounding polymer segments, contributing to the rise in G′ at high frequency while persisting in the terminal region to suppress the frequency dependence of G′ and produce the early flattening observed in the 20MA series. The progressive development of this interfacial structure with compatibilizer content is examined morphologically for the 2.5MA and 20MA systems in Section 3.4. These talc-associated regions may increase the effective interaction volume of the platelets and, once neighboring regions overlap, develop into deformable, stress-bearing connections that contribute to elastic continuity across the interparticle space [29,30,32,33,34,35,36]. As discussed quantitatively in Section 3.5, effective interaction-volume expansion and elastomer-mediated coupling should therefore be regarded as successive and interdependent stages of the same connectivity process rather than as separate competing mechanisms.

The Cole–Cole plots in Figure 4c and Figure 5c provide further insight into the frequency-domain viscoelastic spectrum associated with network development in the 10MA and 20MA composites. For the 10MA series, the samples with lower talc contents, 10MA-T5 and 10MA-T10, display semi-circular arcs, characteristic of systems governed predominantly by the viscoelastic properties of the polymer matrix. This response reflects a relatively homogeneous relaxation-time distribution, consistent with PP-dominated behavior in the absence of extensive filler structuring [24,27,42]. As the talc content increases to 20 wt% and beyond within the same series, the Cole–Cole curves deviate markedly from the ideal semi-circle, exhibiting elongated tails extending toward lower frequencies. This distortion indicates the emergence of long-time relaxation modes associated with filler-induced constraints and emerging network connectivity [24,27,42]. The loss of the semi-circular shape at higher talc loadings therefore highlights a transition from matrix-dominated behavior toward an increasingly filler-constrained response, in agreement with the percolation behavior identified from the G′ and tan δ analyses. The influence of compatibilizer content becomes even more pronounced in the 20MA series (Figure 5c), where the tail-dominated Cole–Cole response appears across all talc contents, including 20MA-T5. Notably, even at 5 wt% talc, the plots show only a faint trace of a semi-circular arc, with extended low-frequency tails already dominating the profile. This shift indicates that the relaxation response is no longer governed solely by the PP matrix but is consistent with an increasing contribution from talc-associated EPDM-g-MA-rich regions. As the talc content increases, the tail becomes further accentuated, without the emergence of secondary arcs or discontinuities that would suggest a distinct incompatible phase contribution. Instead, the uniform evolution of the Cole–Cole shapes is consistent with a relatively continuous relaxation response across the PP/EPDM-g-MA/talc system rather than a separate macroscopic phase-separated relaxation process. The increasing contribution associated with talc and EPDM-g-MA at 20 wt% compatibilizer therefore highlights the coupled effect of talc and compatibilizer content on the relaxation-time distribution. These features are consistent with the elastomer-mediated network interpretation proposed above.

Stress relaxation behavior of PP/talc composites containing 10 wt% and 20 wt% EPDM-g-MA is shown in Figure 4d and Figure 5d, respectively. The results reveal a stepwise improvement in the long-time stress-bearing capacity of the composites with increasing compatibilizer content. Compared with the lower-compatibilizer systems, the 10MA series exhibits a stronger elastic contribution, while the 20MA series shows further enhancement in both stress retention and network stability. These trends are consistent with a gradual transition from transient, weakly connected structures toward more persistent elastomer-mediated connectivity as the EPDM-g-MA content is increased [27,29,30,32,33,34,35,36]. In the 10MA series (Figure 4d), the 10MA-T5 and 10MA-T10 samples exhibit rapid stress decay, indicating that the network structure remains relatively weak and transient at low talc contents. A marked change occurs at 10MA-T20, where the relaxation curve begins to flatten and shows a clear long-τ tail, consistent with the percolated response identified above. At 30 and 40 wt% talc, the relaxation behavior transforms into a power-law-like decay with no abrupt drops in modulus, supporting the presence of a persistent rheologically connected structure over the experimental time window. In the 20MA series (Figure 5d), even the lowest talc content sample, 20MA-T5, exhibits high residual stress with very slow relaxation, consistent with the early onset of the proposed elastomer-mediated network response. This trend intensifies at 20MA-T10 and 20MA-T30, where the E(t) curves maintain high values over extended time scales with no signs of collapse, indicating a highly elastic and well-connected system. A distinct behavior appears in the 20MA-T40 sample. The stress level at short times is the highest among all formulations, indicating that the network is extremely rigid. At longer times, however, the curve drops sharply, revealing a long-time stress collapse. Unlike both the uncompatibilized T40 system and the 2.5MA-T40/5MA-T40 blends, the 20MA-T40 formulation combines the highest initial stress level with a subsequent loss of long-time stability. This behavior suggests that the collapse may be associated with an excessively constrained or over-connected structure rather than insufficient initial connectivity. As interparticle connections increase and the available pathways for viscoelastic flow diminish, the network may become less able to redistribute stress through chain mobility. Localized stress may then build up during relaxation, leading to an abrupt collapse of the stress-bearing structure. Therefore, the stress relaxation results show that EPDM-g-MA improves the long-time stress-bearing capacity and melt-state network stability of the PP/talc system, but excessive compatibilizer content at very high talc loading may produce an overly rigid network that loses stability under prolonged deformation.

### 3.2. Isolating the Elastomer-Mediated Bridging Mechanism Using PP-g-MA and Hybrid Reference Systems

To distinguish the EPDM-g-MA response from conventional MA-mediated interfacial modification and from the independent contribution of an elastomeric phase, PP-g-MA and hybrid PP-g-MA/EPDM reference systems were examined. The hybrid formulations were designed to match the corresponding EPDM-g-MA systems in total MA and elastomer contents, with 2.5PPMA–2.5EPDM and 20PPMA–20EPDM serving as counterparts to the 2.5 and 20 wt% EPDM-g-MA formulations, respectively. The reference systems were evaluated using the same low-frequency G′ and tan δ criteria applied in Section 3.1. This comparison tests whether the early network formation observed for EPDM-g-MA requires covalent integration of the MA functionality and elastomeric backbone within the same compatibilizer chain.

#### 3.2.1. PP-g-MA Systems

**Figure 6:**
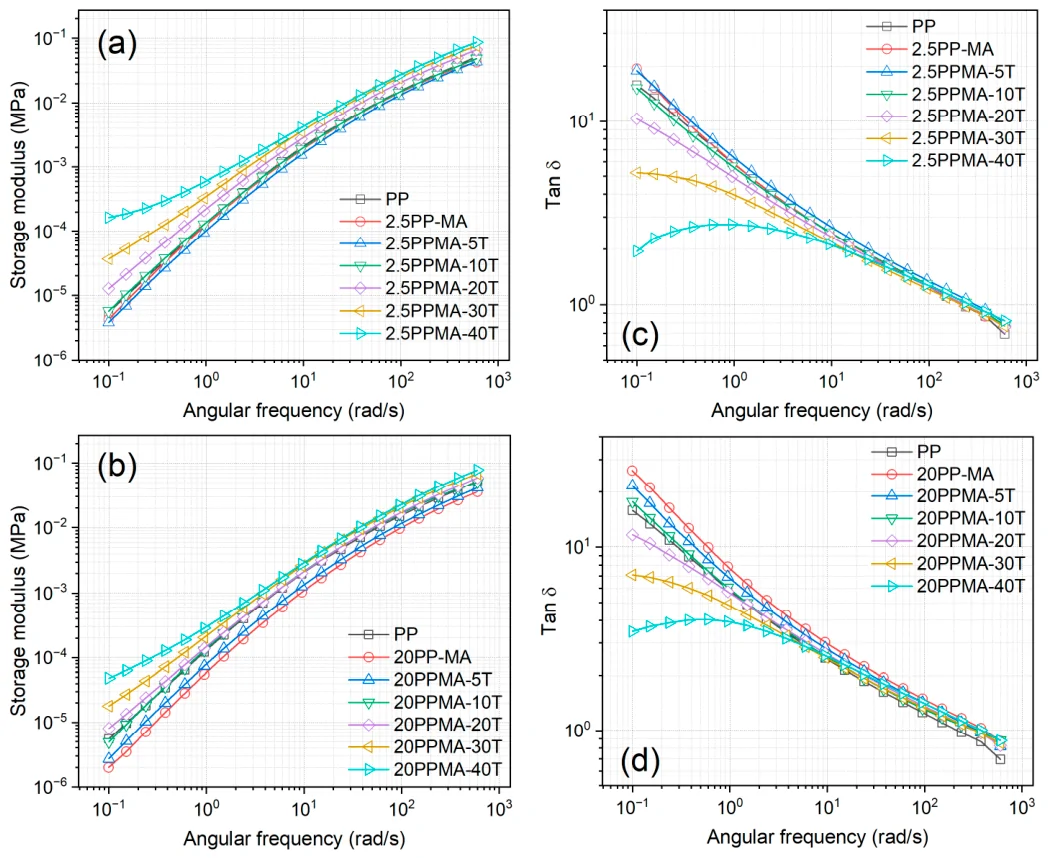
Rheological characterization of PPMA-series composites: (**a**) G′ versus ω for the 2.5PPMA series; (**b**) G′ versus ω for the 20PPMA series; (**c**) tan δ versus ω for the 2.5PPMA series; (**d**) tan δ versus ω for the 20PPMA series.

PP-g-MA was examined at 2.5 and 20 wt%, the lowest and highest EPDM-g-MA contents studied in Section 3.1, enabling a direct one-to-one comparison with the corresponding EPDM-g-MA formulations without the need for intermediate compositions (Figure 6). The rheological behavior of these systems was evaluated using the same frequency-sweep criteria defined above. The G′ values of the PP-g-MA compatibilized series at high frequency (≈600 rad s^−1^) exhibit a notable increase relative to the uncompatibilized T-series at the same talc loading (Figure 6a,b). It is important to note that the modulus values of the neat PP-g-MA/PP blends, without talc, are slightly lower than that of neat PP. This behavior contrasts with the intrinsic elastic contribution observed for the 20MA talc-free blend, indicating that PP-g-MA alone does not introduce an additional elastic component into the melt. However, when talc is incorporated, the high-frequency modulus clearly increases compared with the corresponding uncompatibilized composites. This increase becomes more pronounced as the PP-g-MA content is raised from 2.5 to 20 wt%, indicating that PP-g-MA improves filler–matrix interfacial interactions under rapid deformation [4,5,11]. These results confirm the presence of a compatibilization effect mediated by PP-g-MA. At low angular frequencies, the G′ level rises modestly with talc content, which is consistent with improved talc dispersion; however, this rise is not accompanied by a flattening of the terminal slope, indicating that no rheologically connected filler network is detected. This absence of a low-frequency G′ plateau persists even at 40 wt% talc (Figure 6a,b), where the uncompatibilized T-series had shown a well-defined terminal plateau consistent with percolation. By contrast, the PP-g-MA-modified T40 compositions do not fully recover this plateau-like response, suggesting that PP-g-MA does not lower the percolation threshold and may even delay the formation of a system-spanning talc network through increased particle separation associated with improved dispersion. This behavior is consistent with the conventional expectation that improved dispersion increases the mean interparticle distance and therefore maintains, or slightly raises, the filler content required for direct particle connectivity [25,32,36].

The tan δ responses show the same trend (Figure 6c,d). The terminal tan δ response remains frequency-dependent across the PP-g-MA compositions, without a systematic approach to the near-zero slope characteristic of the critical regime [19,20,21,22,24,27]. This response differs from that of the EPDM-g-MA compatibilized systems, where the terminal slope of G′ decreases much earlier and more strongly with increasing talc loading. In the PP-g-MA formulations, the terminal slope also decreases with talc content, for example from 1.423 at 2.5PPMA-T5 to 0.567 at 2.5PPMA-T40 and from 1.468 at 20PPMA-T5 to 0.790 at 20PPMA-T40. However, these reductions remain less pronounced than those observed in the EPDM-g-MA series, particularly at low talc contents. Therefore, the characteristic early network formation observed in EPDM-g-MA systems cannot be explained by MA-driven interfacial adhesion alone under the conditions examined. It may instead depend on either the integration of MA functionality onto an elastomeric backbone or the presence of an elastomer phase alongside MA functionality. This distinction is examined further using the hybrid PP-g-MA/EPDM systems in Section 3.2.2.

#### 3.2.2. Hybrid PP-g-MA/EPDM Systems

**Figure 7:**
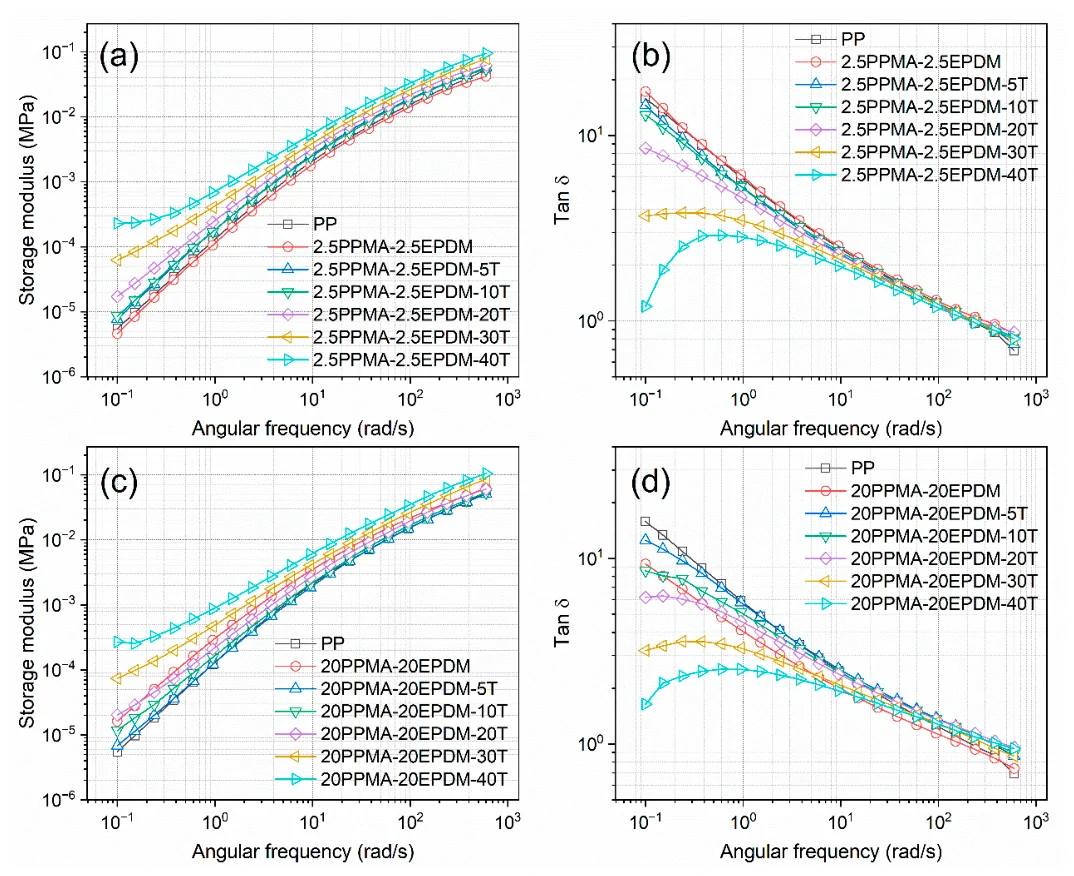
Rheological characterization of hybrid PPMA/EPDM-series composites: (**a**) G′ versus ω and (**b**) tan δ versus ω for the 2.5PPMA–2.5EPDM series; (**c**) G′ versus ω and (**d**) tan δ versus ω for the 20PPMA–20EPDM series.

The PP-g-MA results showed that MA functionality alone did not reproduce the early percolation behavior of EPDM-g-MA, indicating that simple interfacial adhesion is insufficient to explain the observed response. The hybrid PP-g-MA/EPDM systems were designed to determine whether MA functionality and the elastomer phase must be covalently integrated within the same chain, or whether their coexistence as separate macromolecular components is sufficient to generate comparable network connectivity. Because PP-g-MA and EPDM-g-MA have comparable MA grafting levels, the hybrid blends were matched to the corresponding EPDM-g-MA formulations in terms of total MA and elastomer content, providing a direct test that decouples the MA functionality from the elastomer backbone (Figure 7).

The hybrid PP-g-MA/EPDM systems reveal a behavior that is best understood by considering the G′ and tan δ responses together. In the G′ curves (Figure 7a,c), the low-frequency modulus of both 2.5PPMA–2.5EPDM and 20PPMA–20EPDM rises only moderately with talc content, and the terminal slope remains frequency-dependent across most compositions; a clear low-frequency plateau develops only at the highest talc loadings. The tan δ curves (Figure 7b,d), however, indicate an apparently earlier transition. In the 2.5PPMA–2.5EPDM series, the low-frequency slope of tan δ approaches zero at approximately 30 wt% talc, and in the 20PPMA–20EPDM series this apparent transition shifts to around 20 wt% talc—both earlier than the 40 wt% threshold of the uncompatibilized T-series. The two indicators therefore do not coincide: tan δ suggests a network-like transition at 20–30 wt% talc, whereas G′ shows no corresponding terminal plateau at these loadings.

This decoupling is itself informative. In the EPDM-g-MA systems, the onset of percolation is marked by the simultaneous flattening of both G′ and tan δ, consistent with a stress-bearing rheological network [19,20,21,22,24,27]. In the hybrid systems, by contrast, the flattening of the tan δ slope is not accompanied by a rise in low-frequency G′. Since tan δ = G″/G′, a decrease in tan δ without any increase in G′ indicates that the change is dominated by a reduction in viscous dissipation rather than by a corresponding growth in elasticity. This reduced dissipation may arise from the substantial fraction of relatively low-loss elastomer introduced by the unmodified EPDM, which lowers the terminal-region tan δ without establishing continuous, load-bearing connectivity between talc platelets. The weak, non-covalent interactions between the unmodified EPDM chains and the PP-g-MA segments associated with the talc surface likely provide a limited elastic contribution, but appear insufficient to tether adjacent platelets in a persistent manner [29,30,32,33,34,35,36]. Consequently, the hybrid blends display only a partial, apparent enhancement of terminal elasticity and lack the fully developed, bridging-driven network characteristic of EPDM-g-MA. In the EPDM-g-MA systems, the covalent integration of MA functionality onto the elastomer backbone is associated with a simultaneous G′ plateau and tan δ suppression already at 5 wt% talc. This comparison demonstrates that the mere coexistence of MA functionality and an elastomer phase is insufficient under the present formulation and processing conditions; instead, covalent integration within the same chain appears to be a key structural requirement for generating the proposed elastomer-mediated bridging network. The substantially different melt-flow characteristics of PP-g-MA and EPDM-g-MA may contribute to differences in their absolute viscoelastic response and are therefore not disregarded. However, melt-flow characteristics alone do not account for the observed network transition: the unmodified EPDM used in the hybrid systems has a melt index comparable in magnitude to that of EPDM-g-MA, yet the hybrid PP-g-MA/EPDM systems do not reproduce the pronounced low-threshold response of EPDM-g-MA. Accordingly, the present comparison is interpreted as evidence that compatibilizer architecture plays a major role in the network-forming response, rather than as proof that molecular-weight-related effects are completely absent.

### 3.3. Strain Sweep Test

The increasing elastic character observed in the frequency–sweep analysis raises an important question regarding whether the resulting structure remains stable under increasing deformation or becomes highly strain sensitive. Strain–sweep measurements were therefore used to probe the transition from linear to nonlinear viscoelastic behavior and to compare the resistance of the different microstructures to deformation-induced breakdown [29,30,31,42]. The complete strain–sweep curves for all formulations are provided in Figure S1 of the Supplementary Materials. Although this test does not directly quantify melt strength, it provides an indirect measure of melt-state structural robustness and deformation resistance [29,30,31,42]. Two parameters were extracted from the G′–strain response. The plateau modulus (G′_p_) quantifies the small-strain elastic rigidity of the melt microstructure and, in network-forming systems, is sensitive to the degree of structural confinement imposed by compatibilizer–filler interactions. The critical strain (γc) was defined as the strain at which G′ decreased by 10% from G′_p_, marking the onset of nonlinear deformation and indicating the strain tolerance of the underlying microstructure. These parameters were determined for the EPDM-g-MA, PP-g-MA, and hybrid PP-g-MA/EPDM systems, enabling direct comparison of network rigidity and strain sensitivity across all compatibilization strategies.

**Figure 8:**
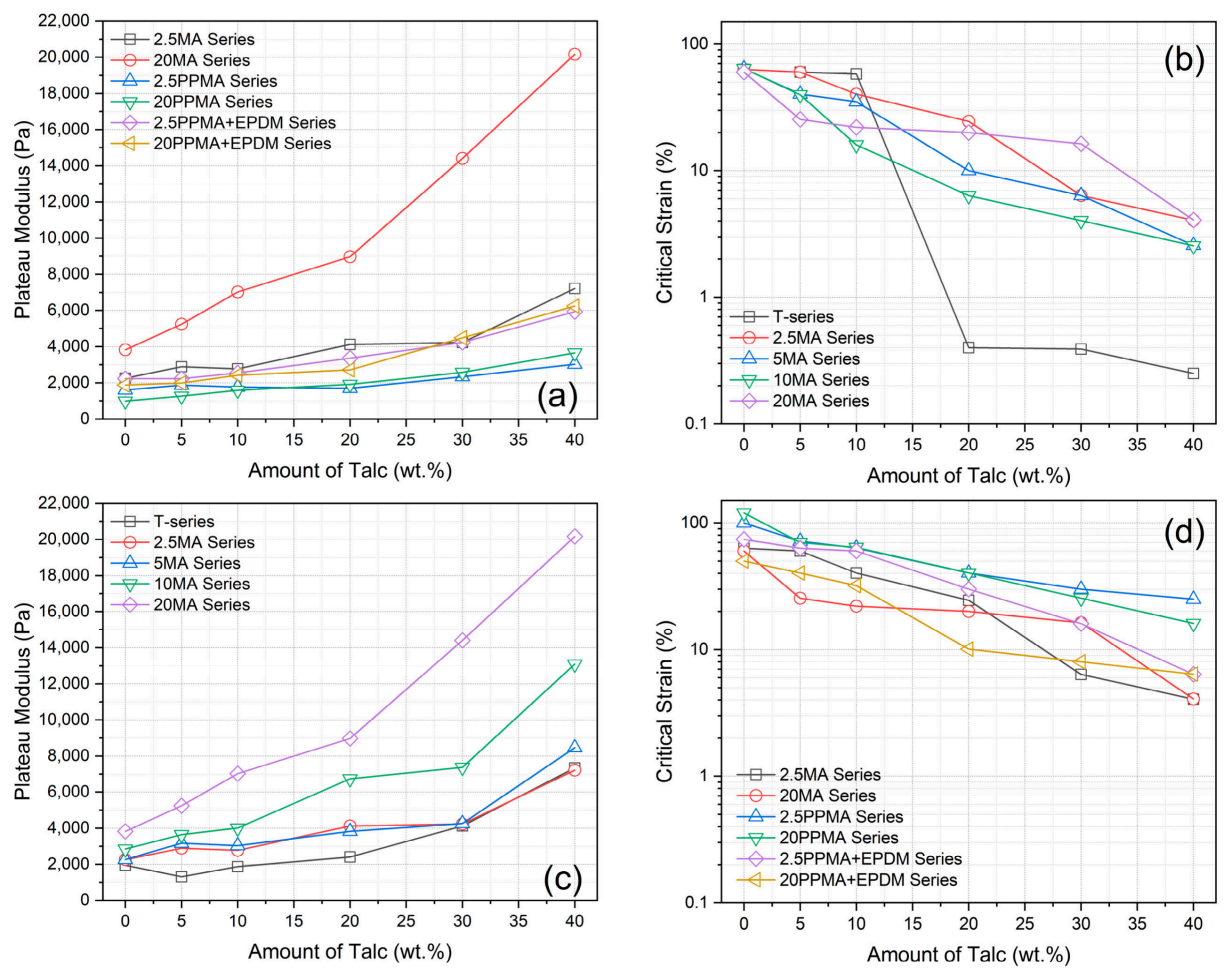
Comparison of strain sweep parameters across all sample series; (**a**,**c**) plateau modulus values and (**b**,**d**) critical strain values.

The plateau modulus data in Figure 8a,c reveal a clear rigidity hierarchy among the EPDM-g-MA, PP-g-MA, and hybrid PP-g-MA/EPDM architectures. The EPDM-g-MA-modified blends exhibit the largest increase in G′_p_ with increasing talc content, reaching approximately 20,000 Pa for 20MA-T40, consistent with strong graft-mediated confinement and the development of elastomer-mediated connectivity between talc particles. In the absence of talc, only the 20MA blend displays a distinctly elevated G′_p_, whereas 10MA remains comparable to the lower-compatibilizer series, indicating that a substantial intrinsic elastic contribution emerges only at the highest EPDM-g-MA content. By contrast, the PP-g-MA systems show only a moderate increase in G′_p_ across the talc range, in line with classical compatibilization through improved interfacial adhesion and dispersion without the formation of a strongly constrained deformation field [4,5,11]. The hybrid PP-g-MA/EPDM formulations remain between these two limits, exhibiting higher rigidity than the PP-g-MA systems but substantially lower G′_p_ values than the corresponding EPDM-g-MA formulations. The resulting order, EPDM-g-MA > hybrid PP-g-MA/EPDM > PP-g-MA, indicates that the pronounced modulus amplification does not arise from the intrinsic elastomeric character of EPDM alone. Instead, it is maximized when the MA functionality and elastomer backbone are covalently integrated within EPDM-g-MA, supporting the development of talc-associated elastomer-rich regions capable of contributing to persistent interparticle coupling. Such connectivity is not reproduced to the same extent by freely dispersed EPDM in the hybrid systems, resulting in a more continuous and strongly confined microstructure in the EPDM-g-MA series.

The critical strain data in Figure 8b,d show the opposite overall ordering and reveal a clear rigidity–strain-tolerance trade-off among the three architectures. The uncompatibilized T-series undergoes the most abrupt reduction in γc, falling below 1% once the talc content exceeds 10 wt%, consistent with weak PP/talc adhesion and early interfacial sliding [8,9,29]. All compatibilized systems retain substantially higher γc values than this baseline. Among them, PP-g-MA generally provides the widest linear viscoelastic range because its less confined microstructure permits greater deformation before the onset of nonlinearity. The hybrid PP-g-MA/EPDM systems exhibit intermediate behavior, whereas the EPDM-g-MA formulations combine the highest G′_p_ values with the lowest γc values among the compatibilized systems, consistent with stronger deformation confinement associated with the proposed elastomer-mediated bridging mechanism [29,30,32,33,34,35,36]. The detailed ranking within the EPDM-g-MA series depends on talc loading. At 5 wt% talc, 20MA exhibits the lowest γc, suggesting that the high EPDM-g-MA content already promotes localized interactions that restrict the deformation field; nevertheless, γc remains above approximately 10%, indicating that the structure is not yet excessively strain-sensitive. At intermediate talc contents of 10–30 wt%, 10MA becomes the least strain-tolerant formulation. At this composition, the increased network rigidity associated with the proposed bridging mechanism appears insufficiently balanced by the elastomer-segment contribution. The 20MA series retains higher γc values over the same range, suggesting that its larger elastomer fraction partially offsets the bridging-associated rigidity, producing a more favorable balance between stiffness and deformation tolerance. At 40 wt% talc, this advantage largely disappears, and the EPDM-g-MA formulations converge to γc values of approximately 2–4%, indicating that the high degree of talc-associated connectivity increasingly governs the onset of nonlinearity.

Taken together, the G′_p_ and γc results support the interpretation that the interparticle connections associated with the unusually low apparent percolation threshold also influence the nonlinear deformation response. The same interparticle connections that increase small-strain rigidity and long-time stress retention restrict the ability of the microstructure to accommodate increasing deformation, revealing a clear rigidity–strain-tolerance trade-off rather than two independent effects. This balance is particularly relevant to the foaming of linear polyolefins such as PP and HDPE, where limited melt strength and insufficient stress-bearing capacity promote cell-wall thinning, coalescence, and collapse during expansion [44,45]. The EPDM-g-MA architecture provides a rheological basis for overcoming this limitation by promoting a stress-retaining, rheologically connected structure at comparatively low talc contents while substantially increasing the plateau modulus. These characteristics indicate a strong potential for improving melt-state support and cellular stability during foaming, although direct confirmation requires extensional rheology and processing-scale foam experiments. More broadly, the architecture-dependent balance between rigidity and deformation tolerance provides a basis for tailoring compatibilizer design to other processing routes, including sheet and film extrusion, fiber spinning, and injection molding, where different levels of melt support and deformability are required.

### 3.4. Morphological Evidence for the Elastomer-Mediated Bridging Mechanism

Scanning electron microscopy was employed as a complementary morphological technique to examine whether the fracture-surface characteristics of PP/talc composites are consistent with the structural differences inferred from rheological measurements. While rheological tests sensitively reveal changes in melt-state viscoelastic response, SEM provides direct information on filler distribution, interfacial integrity, and fracture morphology, which are critical for assessing the morphological basis of the proposed elastomer-mediated bridging mechanism [8,9]. The SEM analysis was therefore conducted on all 20 wt% talc-containing formulations, enabling the effect of compatibilizer type and concentration to be evaluated at a fixed filler content, independently of variations in talc loading.

**Figure 9:**
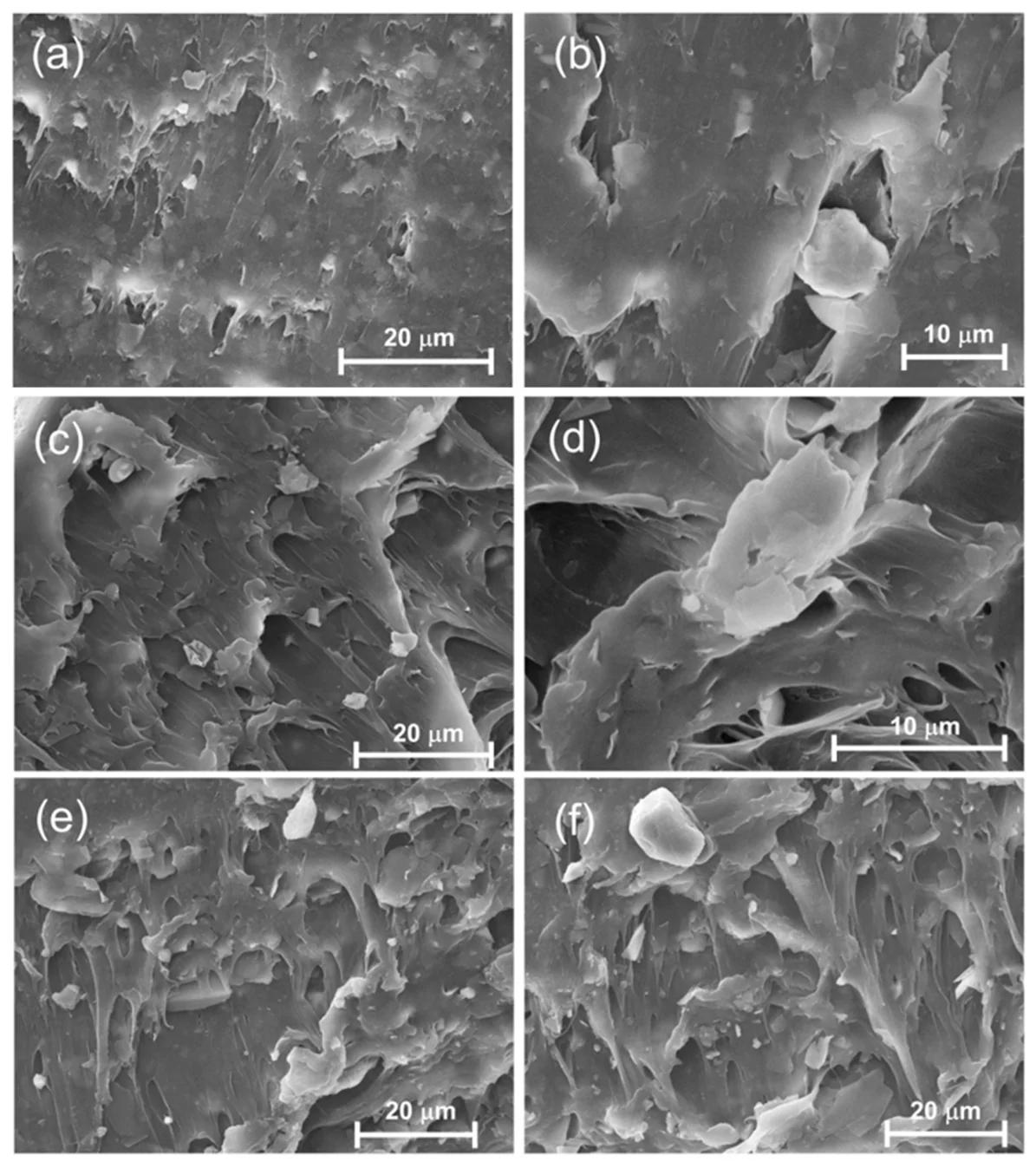
SEM micrographs of (**a**,**b**) the uncompatibilized T20 composite, (**c**,**d**) the 2.5PPMA–T20 composite, and (**e**,**f**) the 20PPMA–T20 composite at two magnifications.

The fracture-surface morphology of the uncompatibilized T20 composite is presented in Figure 9a,b. The low-magnification image (Figure 9a) reveals talc platelets distributed throughout the PP matrix, with visible lateral dimensions ranging approximately from 2 to 10 µm. The morphology is characterized by clearly exposed talc surfaces and sharply defined platelet edges, which are consistent with limited coverage of the filler surface by the polymer phase. This feature is more evident in the higher-magnification image, where the talc platelets appear largely uncoated. A further characteristic of the T20 morphology is the presence of discontinuities along the talc–matrix boundary. Isolated interfacial gaps and pull-out cavities are consistent with insufficient adhesion between the filler and the matrix [8,9]. The exposed filler surfaces and interfacial voids are consistent with preferential crack propagation along the weak particle–matrix interface, supporting a predominantly interfacial (adhesive) failure mode [8,9]. In addition, the matrix exhibits a relatively smooth and featureless fracture surface, with no pronounced evidence of ductile drawing or fibrillation. These morphological features support the presence of a weakly coupled PP/talc interface and provide the baseline structure against which the effects of the different interfacial-modifier architectures can be evaluated.

Figure 9c,d and Figure 9e,f present the fracture-surface morphologies of 2.5PPMA–T20 and 20PPMA–T20, respectively. These PP-g-MA systems provide morphological benchmarks for distinguishing conventional MA-mediated interfacial modification from the elastomer-grafted architecture of EPDM-g-MA. In the 2.5PPMA–T20 composite, the visible talc platelets remain within the micrometre scale, with lateral dimensions predominantly in the range of approximately 2–10 µm, similar to the uncompatibilized T20 sample. However, the interfacial morphology differs markedly from that of T20. Although the talc surfaces remain largely distinguishable and no continuous interfacial layer is evident around the platelet surfaces, the interfacial gaps previously observed along the talc–matrix boundary are substantially suppressed. This morphology is consistent with improved local continuity of the filler–matrix interface without the formation of an enveloped platelet morphology. The comparison between the higher-magnification images of T20 and 2.5PPMA–T20, Figure 9b and Figure 9d, respectively, further highlights the reduction in interfacial debonding and pull-out cavities, supporting the rheological interpretation that PP-g-MA primarily enhances classical filler–matrix adhesion [4,5,11]. The effect of increasing PP-g-MA content is further examined in Figure 9e,f for the 20PPMA–T20 formulation. This high PP-g-MA level was not selected as a practically optimized formulation, but rather to probe the upper limit of the conventional MA-mediated interfacial modification route and to establish a clear benchmark against the EPDM-g-MA architecture. Even at this elevated PP-g-MA content, the talc platelets do not appear to be enveloped by a continuous interfacial phase. Instead, the morphology reflects improved interfacial continuity and a more homogeneous distribution of talc within the polymer phase, with the filler–matrix boundary remaining comparatively well integrated. In addition, finer visible talc domains are more frequently observed in the 20PPMA–T20 morphology, suggesting a possible improvement in dispersion or partial breakup of larger talc aggregates during melt compounding. This apparent improvement in dispersion is consistent with the low-frequency increase in G′ identified from the rheological measurements (Section 3.2.1); however, the PP-g-MA series does not exhibit the marked downward shift in percolation threshold observed for EPDM-g-MA systems. Therefore, the enhanced dispersion and improved interfacial adhesion provided by PP-g-MA are insufficient to generate the elastomer-mediated network connectivity observed in the EPDM-g-MA systems. This observation is regarded as qualitative morphological evidence rather than a quantitative measure of particle-size reduction and reinforces that filler dispersion alone is not the governing factor behind the amplified elastic response of the EPDM-g-MA systems. This distinction becomes important in the comparison with the EPDM-g-MA series discussed below.

**Figure 10:**
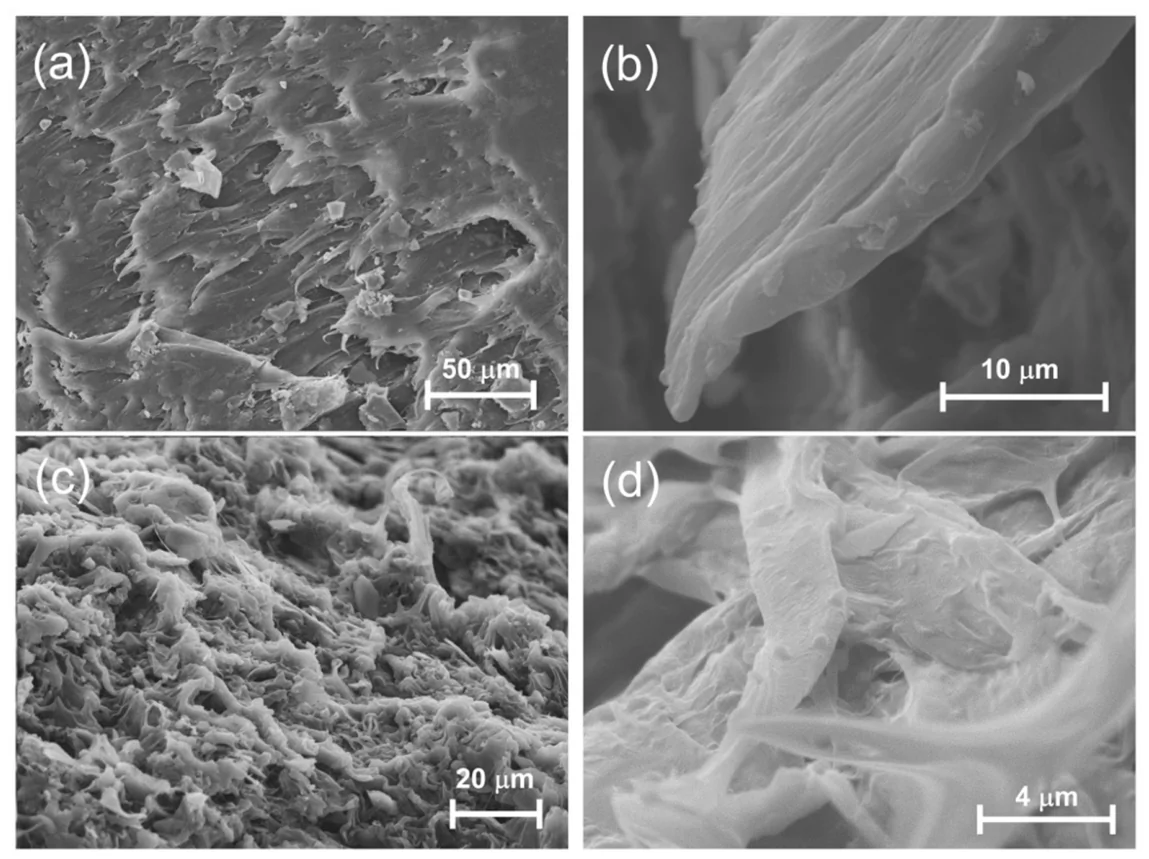
SEM fracture-surface micrographs of PP/talc composites containing 20 wt% talc and compatibilized with (**a**,**b**) 2.5 wt% EPDM-g-MA and (**c**,**d**) 20 wt% EPDM-g-MA, shown at two magnifications.

Figure 10a,b and Figure 10c,d present the fracture-surface morphologies of 2.5MA–T20 and 20MA–T20, respectively. These EPDM-g-MA-containing formulations were examined to assess how the elastomer-grafted MA architecture modifies the PP/talc microstructure at a fixed talc content and to compare its morphological consequences with the PP-g-MA benchmarks discussed above. In both EPDM-g-MA systems, the visible talc platelets remain within the micrometre scale, with lateral dimensions broadly comparable to those observed in T20 and PP-g-MA-containing formulations. Therefore, the main morphological distinction introduced by EPDM-g-MA is not a pronounced change in the apparent talc size range, but rather a substantial alteration of the talc–matrix interfacial region. In the 2.5MA–T20 composite, shown in Figure 10a,b, the talc platelets no longer display the clean and sharply exposed surfaces characteristic of the unmodified T20 sample. Instead, portions of the platelet surfaces appear covered by a thin, discontinuous interfacial layer, and the platelet edges become less sharply defined. Interfacial gaps, debonding voids, and pull-out cavities, which were clearly observed in T20 and only suppressed in the PP-g-MA systems, are largely suppressed in this morphology. The platelets therefore remain more closely associated with the surrounding polymer phase, which is consistent with improved interfacial integrity in the presence of EPDM-g-MA [8,9,13]. The interfacial layer observed around the talc platelets is attributed, based on the formulation, to preferential localization of EPDM-g-MA at the talc surface; however, this assignment should be regarded as a morphology-based interpretation rather than a direct compositional identification. The morphological effect becomes considerably more pronounced in the 20MA–T20 formulation, shown in Figure 10c,d. At this higher EPDM-g-MA content, the talc platelets appear extensively enveloped by a continuous interfacial phase, and the filler surfaces are no longer observed as clean, uncovered platelets. In contrast to the thinner and discontinuous interfacial layer observed in 2.5MA–T20, the interfacial phase in 20MA–T20 appears more dominant and continuous across the fracture surface. Importantly, this phase appears to extend into the regions between adjacent talc platelets, suggesting local phase continuity across neighboring filler domains. The surrounding matrix also exhibits a more deformed and ductile fracture texture, in contrast to the smooth and featureless fracture surface of the uncompatibilized T20 composite. The absence of exposed platelet surfaces, interfacial voids, and pull-out features is consistent with a shift from predominantly interfacial crack propagation toward a more cohesive failure path within the enveloping interfacial region [8,9].

The comparison between 2.5MA–T20 and 20MA–T20 reveals a clear concentration-dependent morphological evolution. At low EPDM-g-MA content, talc platelets are partially enveloped by a thin, discontinuous interfacial layer, whereas at high EPDM-g-MA content this interfacial layer evolves into a more continuous interfacial phase that extends between neighboring platelets. This behavior contrasts sharply with the PP-g-MA systems, where even an eightfold increase in modifier content improved interfacial continuity but did not produce an enveloped platelet morphology. The distinction indicates that the morphological evolution observed in the EPDM-g-MA series cannot be attributed solely to MA-mediated adhesion. Instead, the elastomeric backbone of EPDM-g-MA appears to play a key role in promoting a continuous interfacial phase around talc platelets. These SEM observations are consistent with the rheological behavior discussed in Section 3.1, where EPDM-g-MA caused a marked downward shift in the apparent percolation threshold and produced a strongly amplified low-frequency elastic response. The development of a more continuous interfacial morphology around talc platelets and its apparent extension between neighboring filler domains can increase the effective volume of the filler-associated structure, thereby contributing to the emergence of network-like behavior at lower actual talc contents [32,33,34,35,36]. Accordingly, the SEM results provide morphological support for the proposed elastomer-mediated network interpretation, while avoiding the assumption that connectivity arises from direct particle contact alone. The distinction between PP-g-MA and EPDM-g-MA established here provides the morphological basis for comparison with the hybrid PP-g-MA/EPDM systems discussed below.

**Figure 11:**
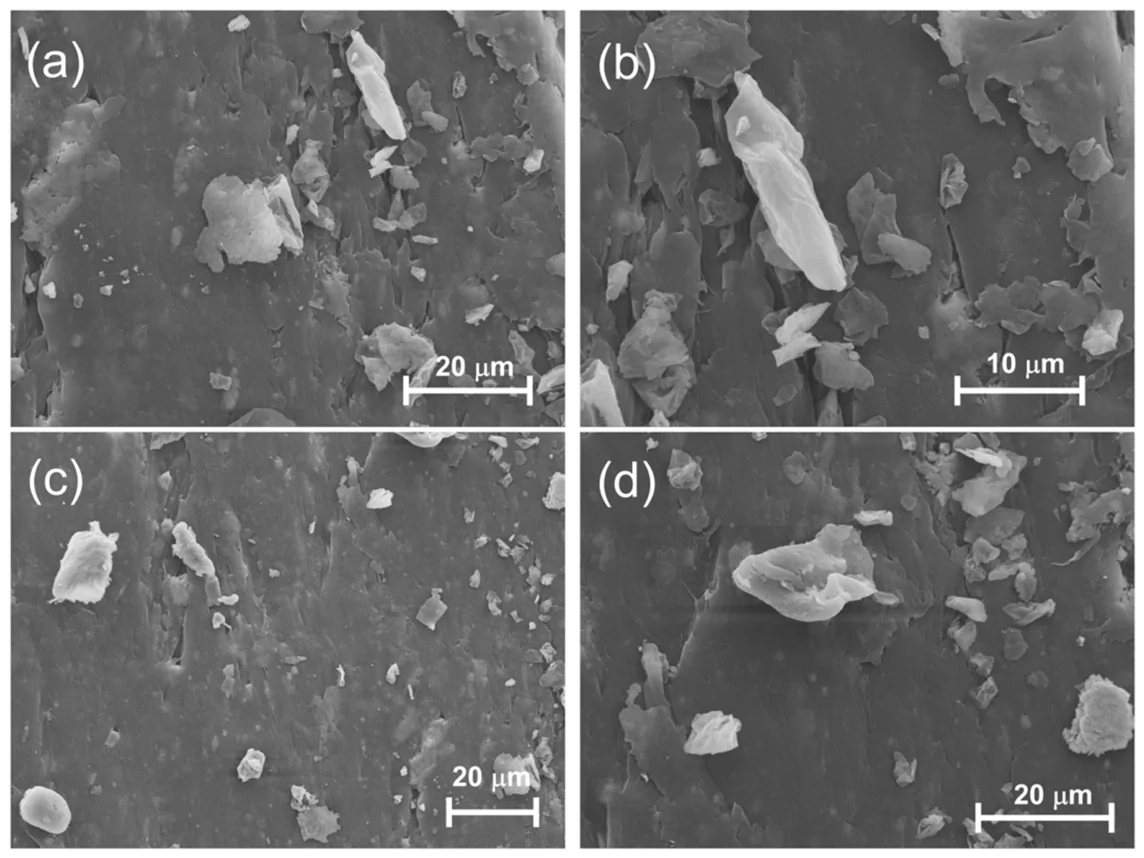
SEM fracture-surface micrographs of the 2.5PPMA–2.5EPDM–T20 composite (**a**,**b**) and the 20PPMA–20EPDM–T20 composite (**c**,**d**), shown at two magnifications.

The fracture-surface morphologies of the hybrid PP-g-MA/EPDM systems are presented in Figure 11a,b and Figure 11c,d for 2.5PPMA-2.5EPDM-T20 and 20PPMA-20EPDM-T20, respectively. In the 2.5PPMA-2.5EPDM-T20 sample, the talc platelets remain clearly exposed, with sharply defined surfaces and edges resembling the uncompatibilized T20 and PP-g-MA morphologies more closely than the corresponding EPDM-g-MA composite. Increasing the hybrid content to 20PPMA-20EPDM produces greater matrix deformation and a more pronounced elastomeric contribution within the surrounding matrix; however, this response is consistent with bulk toughening by the dispersed elastomer rather than the formation of a talc-localized interfacial phase. The talc surfaces therefore remain largely uncovered, and no continuous interfacial phase enveloping the platelets or extending persistently between neighboring talc domains is evident. This morphology differs markedly from that of 20MA-T20, where the platelets are surrounded by a more continuous elastomer-rich interfacial structure and local continuity between adjacent particles is observed. The absence of a comparable enveloping morphology even at the highest hybrid content indicates that the separate coexistence of MA functionality and an elastomer phase is insufficient to reproduce the talc-associated interfacial structure associated with EPDM-g-MA. Together with the rheological response of the hybrid systems (Figure 7), which did not reproduce the coupled low-frequency G′ and tan δ signatures of early percolation, these observations support the conclusion that covalent integration of MA functionality with the elastomer backbone plays a key role in promoting persistent elastomer-mediated interparticle coupling.

### 3.5. Effective Interaction Volume and the Development of Elastomer-Mediated Connectivity

**Figure 12:**
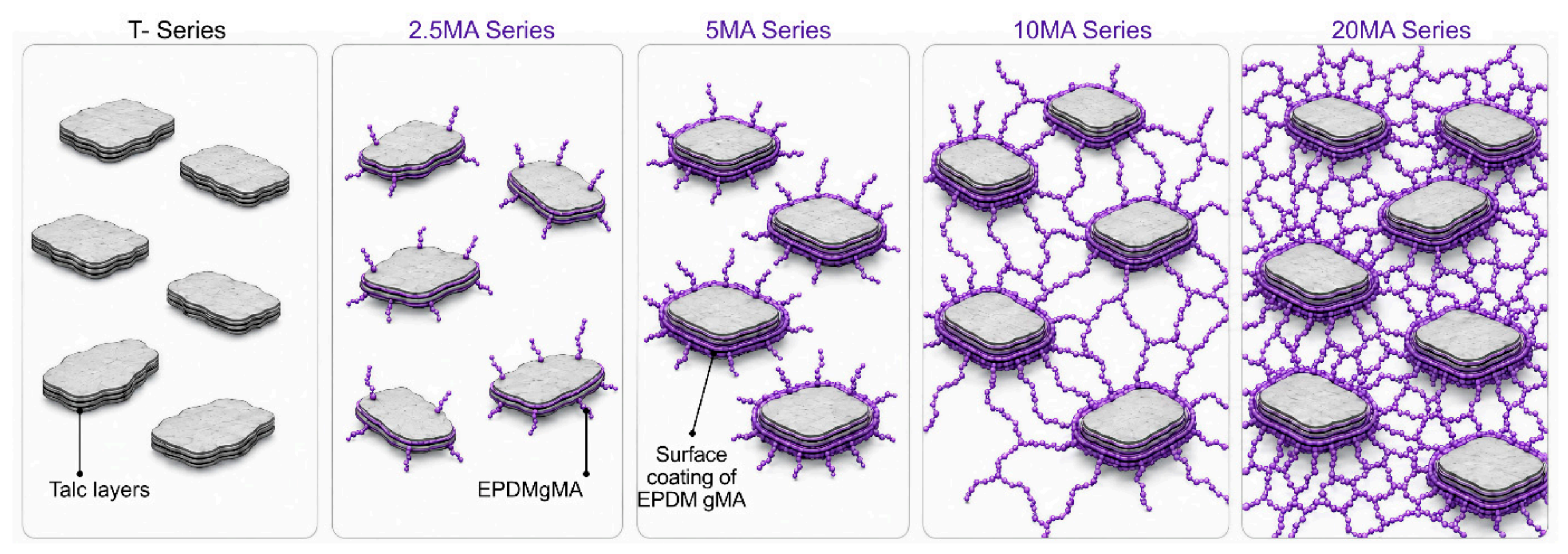
Schematic representation of the proposed evolution of the talc-associated interfacial structure with increasing EPDM-g-MA content.

The apparent rheological network thresholds identified for the EPDM-g-MA series cannot be interpreted solely in terms of the geometric volume occupied by the talc platelets. The uncompatibilized T-series provides a reference state in which long-range connectivity develops only at high talc content, whereas increasing EPDM-g-MA content progressively shifts the network-like response toward substantially lower filler loadings. This change indicates that the relevant connectivity unit is no longer limited to the bare talc platelet. Instead, talc-associated EPDM-g-MA regions are expected to extend the spatial range over which neighboring platelets can interact, thereby increasing their effective interaction volume [32,33,34,35,36]. Effective-volume expansion and elastomer-mediated bridging should not, however, be regarded as mutually exclusive mechanisms. The growth of a talc-associated elastomeric region provides the geometric basis for earlier contact or overlap between neighboring interfacial regions. Once such regions begin to overlap, their polymeric character may allow the interaction to develop beyond simple geometric contact and contribute to deformable, stress-bearing coupling between adjacent platelets [29,30,32,33,34,35,36]. The proposed mechanism is therefore considered as a progressive transition from interfacial-region growth and effective interaction-volume expansion to elastomer-mediated interparticle connectivity, rather than as a competition between geometric and macromolecular explanations (Figure 12).

The uncompatibilized T40 composition was used as a reference for estimating the critical volume fraction associated with network formation. Based on the component densities, 40 wt% talc corresponds to a talc volume fraction of approximately 0.18. In contrast, the actual talc volume fractions at the apparent network thresholds of the EPDM-g-MA series are substantially lower. For example, the talc volume fraction of 20MA-T5 is approximately 0.017, which is nearly one order of magnitude below the reference value obtained for T40. The volume occupied by the bare talc platelets is therefore insufficient to account for the early network-like response observed in the high-EPDM-g-MA formulations. The relevant interaction volume may nevertheless be larger than the bare talc volume if a fraction of EPDM-g-MA is preferentially associated with the platelet surfaces. To examine the maximum possible contribution of this effect, an upper-bound estimate was calculated by assigning the entire EPDM-g-MA volume to the talc-associated region. Under this limiting assumption, the effective interaction volume reaches approximately 0.156 for 2.5MA-T30, 0.188 for 5MA-T30, 0.195 for 10MA-T20, and 0.230 for 20MA-T5. These estimates show that the contribution of the talc-associated polymer region becomes increasingly important as the EPDM-g-MA content rises. At the lowest compatibilizer level, even the upper-bound interaction volume remains below the reference critical value, whereas at higher EPDM-g-MA contents it can approach or exceed this value. These calculations do not demonstrate that the entire EPDM-g-MA phase is localized at the talc surface. Rather, they define the maximum geometric contribution that interfacial-region growth could provide. Since the talc-associated fraction of EPDM-g-MA and the thickness of the resulting interfacial region were not independently quantified, the relative contribution of effective interaction-volume expansion cannot be determined precisely from the present data. Nevertheless, the analysis indicates that geometric expansion of the talc-associated region is likely to contribute to the progressive reduction in the apparent network threshold, particularly at the higher EPDM-g-MA contents.

Geometric overlap alone does not necessarily establish a rheologically effective network. Contact between neighboring talc-associated regions may increase the probability of connectivity, but a persistent low-frequency elastic response requires these contacts to transmit stress over time rather than behave as transient or weakly interacting shells [27,29,30]. The rheological results are consistent with the growth and overlap of the interfacial regions becoming mechanically functional as the EPDM-g-MA content increases. The progressive suppression of the terminal frequency dependence of G′, the corresponding reduction in the low-frequency slope of tan δ, and the more persistent stress-relaxation response are collectively consistent with the development of stress-bearing connections between neighboring platelets. Within this framework, elastomer-mediated bridging represents the mechanically active stage of effective interaction-volume expansion. As talc-associated EPDM-g-MA regions grow and begin to overlap, segments belonging to neighboring regions may interpenetrate and interact across the interparticle space, allowing elastic stress to be transferred without requiring direct rigid talc–talc contact [32,33,34,35,36]. This interpretation is consistent with the substantially earlier emergence of network-like behavior in the high-EPDM-g-MA formulations and with the more continuous talc-associated fracture morphology observed by SEM. The present data do not directly quantify the extent of chain interpenetration or entanglement; therefore, these molecular processes should be regarded as a plausible basis for the observed elastic coupling rather than as independently demonstrated structural features. The reference systems further clarify why expansion of the talc-associated interaction volume does not, by itself, determine whether interfacial overlap develops into a mechanically effective connection. PP-g-MA improves local interfacial continuity and may influence the spatial distribution of the talc platelets, yet it does not generate the same early suppression of terminal behavior observed in the EPDM-g-MA series. This indicates that increasing the effective interaction range of the filler through improved interfacial association does not necessarily produce a stress-bearing network unless the surface-associated region can also sustain deformable coupling between neighboring particles [29,30,32,33,34,35,36]. The failure of the hybrid systems to reproduce this low-threshold response further supports compatibilizer architecture as an important factor governing the transition from interfacial-region growth to stress-bearing interparticle coupling.

Taken together, the effective-volume analysis, rheological response, and comparative morphology support a progressive mechanism in which EPDM-g-MA first increases the interaction volume of the talc platelets through surface-associated interfacial-region growth and subsequently enables elastomer-mediated elastic coupling once neighboring regions overlap. The relative contributions of geometric expansion, chain interpenetration, and possible entanglement cannot be separated quantitatively from the present data because the talc-associated fraction and spatial distribution of EPDM-g-MA were not independently measured. Accordingly, the proposed bridging mechanism should be regarded as a mechanistic interpretation consistent with the combined observations rather than as a directly resolved molecular structure.

## 4. Conclusions

This study demonstrates that compatibilizer architecture can shift PP/talc composites from conventional local interfacial modification toward long-range elastomer-mediated connectivity. The uncompatibilized T-series exhibited an apparent rheological network threshold at approximately 40 wt% talc. This threshold decreased to approximately 30 wt% at 2.5–5 wt% EPDM-g-MA, 20 wt% at 10 wt% EPDM-g-MA, and ≤5 wt% at 20 wt% EPDM-g-MA. In contrast, PP-g-MA did not promote an earlier network transition, while hybrid PP-g-MA/EPDM systems reproduced only part of the terminal-response modification observed for EPDM-g-MA. These comparisons show that improved local interfacial continuity or the separate coexistence of MA functionality and an elastomer phase does not reproduce the low-threshold response generated by their integration within the same compatibilizer architecture.

The combined results support a progressive mechanism in which talc-associated EPDM-g-MA regions increase the effective interaction volume of the platelets and enable mechanically functional overlap between neighboring interfacial regions at progressively lower talc contents. The combined rheological and morphological responses are consistent with the resulting transition from local interfacial modification to deformable, stress-bearing interparticle coupling.

The resulting connectivity increased the small-strain elastic rigidity of the composites but also introduced a composition-dependent reduction in strain tolerance, demonstrating that early network formation does not necessarily provide unlimited structural stability. The relative contributions of geometric interaction-volume expansion, chain interpenetration, and possible entanglement could not be separated quantitatively because the fraction and spatial distribution of talc-associated EPDM-g-MA were not independently measured. The proposed bridging mechanism should therefore be regarded as a mechanistic interpretation supported by the combined rheological, morphological, and architectural comparisons rather than as a directly resolved molecular structure. Within this limitation, the study establishes compatibilizer architecture as a means of controlling not only local filler–matrix adhesion but also the interaction range and mechanical continuity of filler-associated regions in polymer melts.

## Data Availability

The original contributions presented in this study are included in the article/Supplementary Material. Further inquiries can be directed to the corresponding author.

## Supplementary Materials

The following supporting information can be downloaded at: https://www.mdpi.com/article/10.3390/polym18192396/s1, Figure S1: Strain–sweep test results of all samples; Figure S2: Time-sweep measurements at 200 °C for representative materials and formulations used to evaluate rheological stability over the experimental time scale.
