# Supplementary material for: Elastomer-Mediated Interparticle Coupling Promotes Low-Threshold Rheological Network Formation in EPDM-g-MA-Compatibilized PP/Talc Composites
Source: Polymers (Basel). 2026 Sep 30;18(19):2396. doi: 10.3390/polym18192396 (PMC13666641; doi:10.3390/polym18192396)
Supplement: Supplementary file 1 [file polymers-18-02396-s001.zip › polymers-4541520-supplementary.pdf]

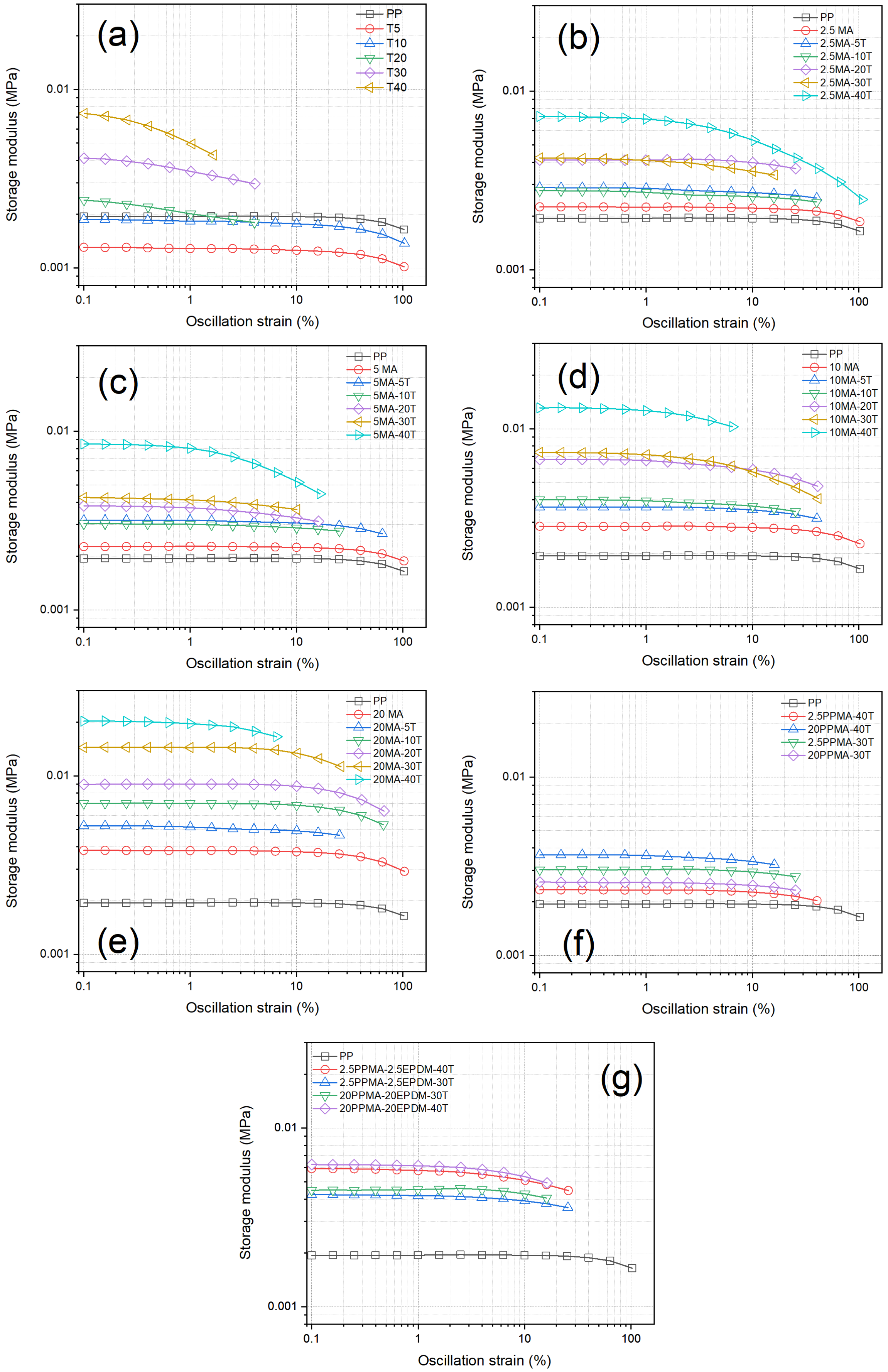

Figure S1. Strain sweep test results of all samples

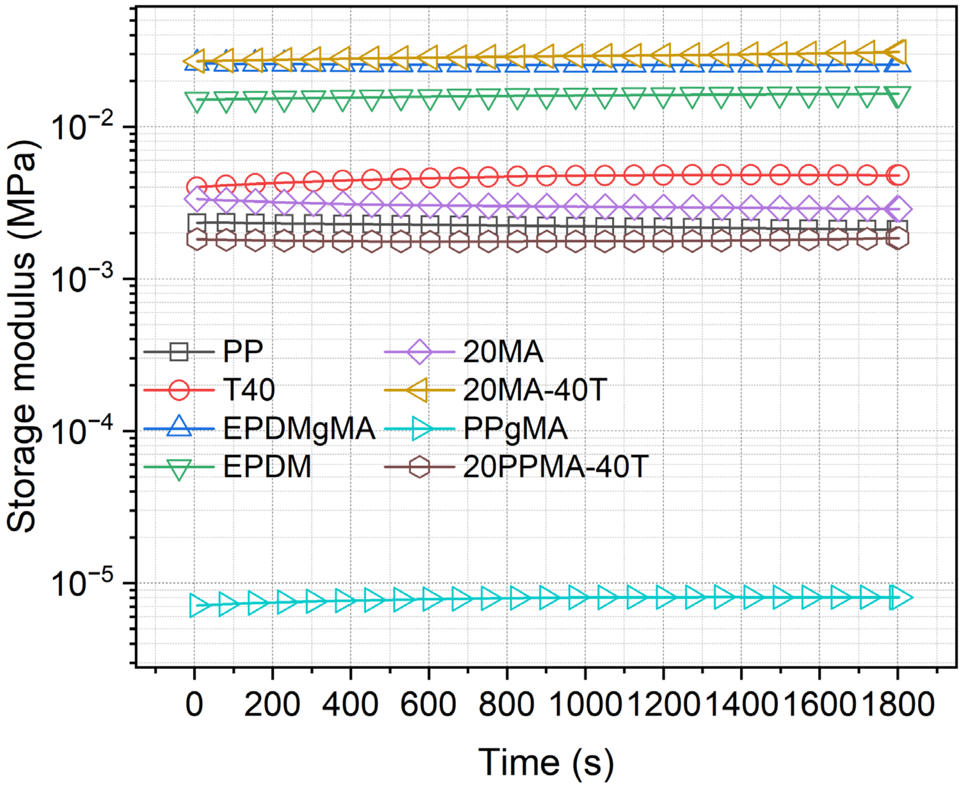

Figure S2. Time-sweep measurements at 200 °C for representative materials and formulations used to evaluate rheological stability over the experimental time scale.
